# Stage-Wise Identification and Analysis of miRNA from Root-Knot Nematode *Meloidogyne incognita*

**DOI:** 10.3390/ijms17101758

**Published:** 2016-10-21

**Authors:** Parthiban Subramanian, In-Chan Choi, Vimalraj Mani, Junhyung Park, Sathiyamoorthy Subramaniyam, Kang-Hyun Choi, Joon-Soo Sim, Chang-Muk Lee, Ja Choon Koo, Bum-Soo Hahn

**Affiliations:** 1 Metabolic Engineering Division, Department of Agricultural Biotechnology, National Institute of Agricultural Sciences, Rural Development Administration, Jeonju 54874, Korea; parthi@chungbuk.ac.kr (P.S.); inchchoi@korea.kr (I.-C.C.); vimal@jbnu.ac.kr (V.M.); hyuny24k@jbnu.ac.kr (K.-H.C.); jssim@korea.kr (J.-S.S.); changmuk@korea.kr (C.-M.L.); 2 Division of Science Education and Institute of Fusion Science, Chonbuk National University, Jeonju 761-756, Korea; jkoo@jbnu.ac.kr; 3 TheragenEtex Bio Institute, Gwanggyo-ro, Suwon 16229, Korea; jhpark98@gmail.com (J.P.); sathiya.moorthy@theragenetex.com (S.S.)

**Keywords:** *Meloidogyne incognita*, stage-specific, microRNA expression, quantitative polymerase chain reaction

## Abstract

In this study, we investigated global changes in miRNAs of *Meloidogyne incognita* throughout its life cycle. Small RNA sequencing resulted in approximately 62, 38, 38, 35, and 39 Mb reads in the egg, J2, J3, J4, and female stages, respectively. Overall, we identified 2724 known and 383 novel miRNAs (read count > 10) from all stages, of which 169 known and 13 novel miRNA were common to all the five stages. Among the stage-specific miRNAs, miR-286 was highly expressed in eggs, miR-2401 in J2, miR-8 and miR-187 in J3, miR-6736 in J4, and miR-17 in the female stages. These miRNAs are reported to be involved in embryo and neural development, muscular function, and control of apoptosis. Cluster analysis indicated the presence of 91 miRNA clusters, of which 36 clusters were novel and identified in this study. Comparison of miRNA families with other nematodes showed 17 families to be commonly absent in animal parasitic nematodes and *M. incognita*. Validation of 43 predicted common and stage-specific miRNA by quantitative PCR (qPCR) indicated their expression in the nematode. Stage-wise exploration of *M. incognita* miRNAs has not been carried out before and this work presents information on common and stage-specific miRNAs of the root-knot nematode.

## 1. Introduction

Root-knot nematodes (RKNs) belong to the group of plant-parasitic nematodes (PPN), which are detrimental to diverse plant species [1]. Members of genus *Meloidogyne* constitute the RKNs, among which *Meloidogyne incognita* (*M. incognita*) is an important agricultural pest [2]. The life cycle of this nematode can be divided into five stages beginning with eggs that develop into root-infecting J2 juveniles. Upon invading the host, the J2 worms attach to the root and develop permanent feeding sites. Molting under favorable conditions leads to the formation of sedentary third (J3) and fourth (J4) stage juveniles. In the next stage, mature male and female adults are formed. Male adults, however, are not required for reproduction and saccate female worms produce eggs that hatch to release J2 worms into the soil to continue infecting neighboring plant roots. *M. incognita* can affect a wide range of hosts and commonly results in stunted plants, loss of vigor, and wilting, symptoms that occur due to the disruption of nutrient and water uptake [1,2]. Conventional control methods against RKNs employ fumigant nematicides and non-fumigant chemicals including carbamates and organophosphates, which are effective but have several drawbacks such as direct environmental pollution, indirect pollution due to mobility from the root zone, and reduction of soil health by adhering to organic matter [3,4]. Currently emerging methods report on the use of RNA interference (RNAi) as an eco-sustainable strategy to control root-knot nematodes from infecting plants.

MicroRNAs (miRNAs) are short endogeneous RNA molecules (length ~22 nt) that are involved in the post-transcriptional regulation of genes in living organisms [5]. Since their discovery, a huge amount of research has been carried out on sequencing miRNAs from several organisms including plants, insects, and higher animals. These RNAs do not code for proteins but are involved in the regulation of mRNA by binding to their 3′ untranslated region (UTR) and causing the phenomenon of RNA interference (RNAi) [6]. As mentioned earlier, RNAi has been widely proposed as a potential strategy to control nematodes in plants [7,8,9,10]. Each miRNA contains a “seed sequence” at its 5′ end that aids target recognition and a stem-loop structure for further processing by enzymes Drosha or Dicer to release a precursor miRNA (pre-miRNA), both properties currently used to identify putative miRNAs [11,12]. Although updated miRNA databases contain huge collections of miRNA from viruses, microalgae, worms, flies, and mammals, their expression in cells and the exact targets of several of these miRNAs remain to be validated [12].

The nematode *Caenorhabditis elagans* provides an excellent model for miRNA-based studies that can be employed to control other parasitic nematodes [13]. Several studies hitherto have been conducted on the development of strategies and identification of miRNA targets to control plant-parasitic nematodes [14,15,16]. In the context of PPNs, the availability of the whole genome sequence of *M. incognita* [17] has led to several investigations focusing on elucidating potential miRNAs that can be used to control the pest in plants. The currently available literature provides us with information on miRNAs in the egg and J2 stages of *M. incognita* [15,18]. Comprehensive information on miRNA of *M. incognita* from all five life stages (egg, J2, J3, J4, and female) would be very useful to understand the physiological processes occurring in the nematode at various stages of its growth and also develop stage-specific markers or control strategies. To our knowledge, stage-wise exploration of miRNAs in *M. incognita* has not been carried out before and this work presents information on the nematode miRNAs expressed at each stage of its life cycle.

## 2. Results

### 2.1. Small RNA Library of Meloidogyne incognita (M. incognita)

**Figure 1:**
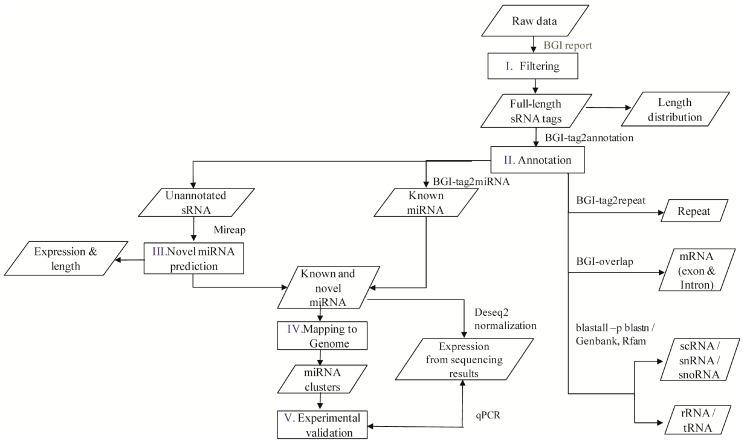
Flowchart of the miRNA analysis performed in the present study. The encircled numerals (I to V) indicate the order in which the experiments were carried out. The sequencing data from an Ilumina sequencer was used as raw data, which were further cleaned and annotated to segregate as well as identify different types of sRNAs. Known miRNAs were identified using miRBase and identification of novel miRNAs was carried out using MIREAP software, results from both of which were then analyzed to study families, clusters, and the validation of their expression.

A flowchart depicting the course of experiments carried out is given in Figure 1. Following deep sequencing, five sRNA libraries of *M. incognita* were constructed to cover the developmental stages of the nematode life cycle with their respective high quality reads, namely: egg (61,825,587), J2 (37,460,733), J3 (38,071,041), J4 (34,295,558), and female stage (39,048,713). From the high-quality reads, further exclusion of non-informative sequences based on PHERD quality, adaptor sequences, contaminated sequences, and sequences containing poly(A) tails was carried out to obtain clean reads (18–30 nt) (Table 1).

**Figure 2:**
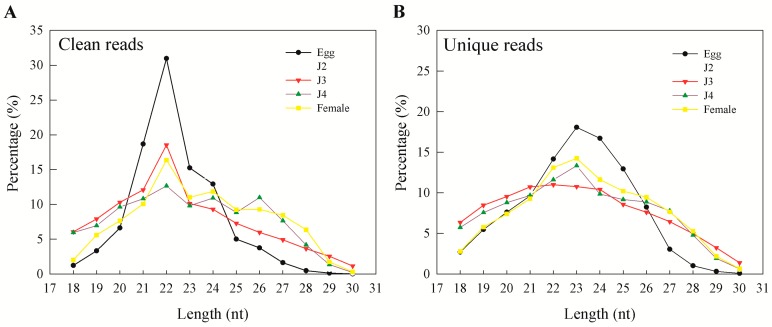
Length distribution of small RNAs at different stages (egg, J2, J3, J4, and female). Frequencies of reads were normalized using DESeq2 to illustrate relative abundances of reads with different lengths under the same scale. (**A**) Clean reads; (**B**) unique reads.

As reads from the different stages had varied sequencing depths, we normalized using the DESeq2 package of R to obtain the DESeq2 values. On studying the length distribution of the sRNAs, the most dominant peaks occurred at 22 nt (Figure 2A). The duplicate reads from the clean read data were removed to obtain unique reads, which were also studied. In the case of unique reads, dominant peaks were observed at 23 nt length for all stages of development (Figure 2B). When we mapped the clean and unique reads to the reference genome (ASM18041v1), we found high mapping ratios in clean read from the egg, J2, and female stages, whereas clean reads from the J3 and J4 stages showed low mapping percentages (Table S1). Unique reads showed low mapping ratios in all the developmental stages.

### 2.2. Classification of M. incognita sRNAs 

**Figure 3:**
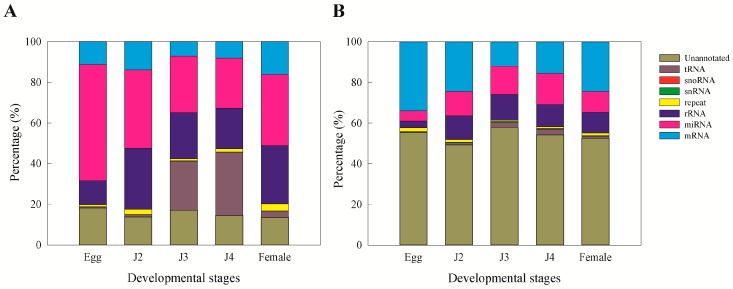
Annotation of small RNAs of *M. incognita*. (**A**) Clean reads; (**B**) unique reads.

The sRNA sequences were annotated by comparing the sequence results to various databases. Among clean reads, we were able to classify 84.55% of the sequences as mRNA, miRNA, rRNA, repeat, snRNA, snoRNA, and tRNA. The remaining 15.45% remained as unannotated sequences (Figure 3A). In the case of the unique reads, a majority of the sRNAs were observed to be unannotated sequences (58.86%) (Figure 3B). Repeat associated RNAs are given in Figure S1. In terms of miRNA, the distribution was high in clean reads but decreased after removal of duplicates (Figure 3B).

### 2.3. Known and Novel miRNAs of M. incognita

**Figure 4:**
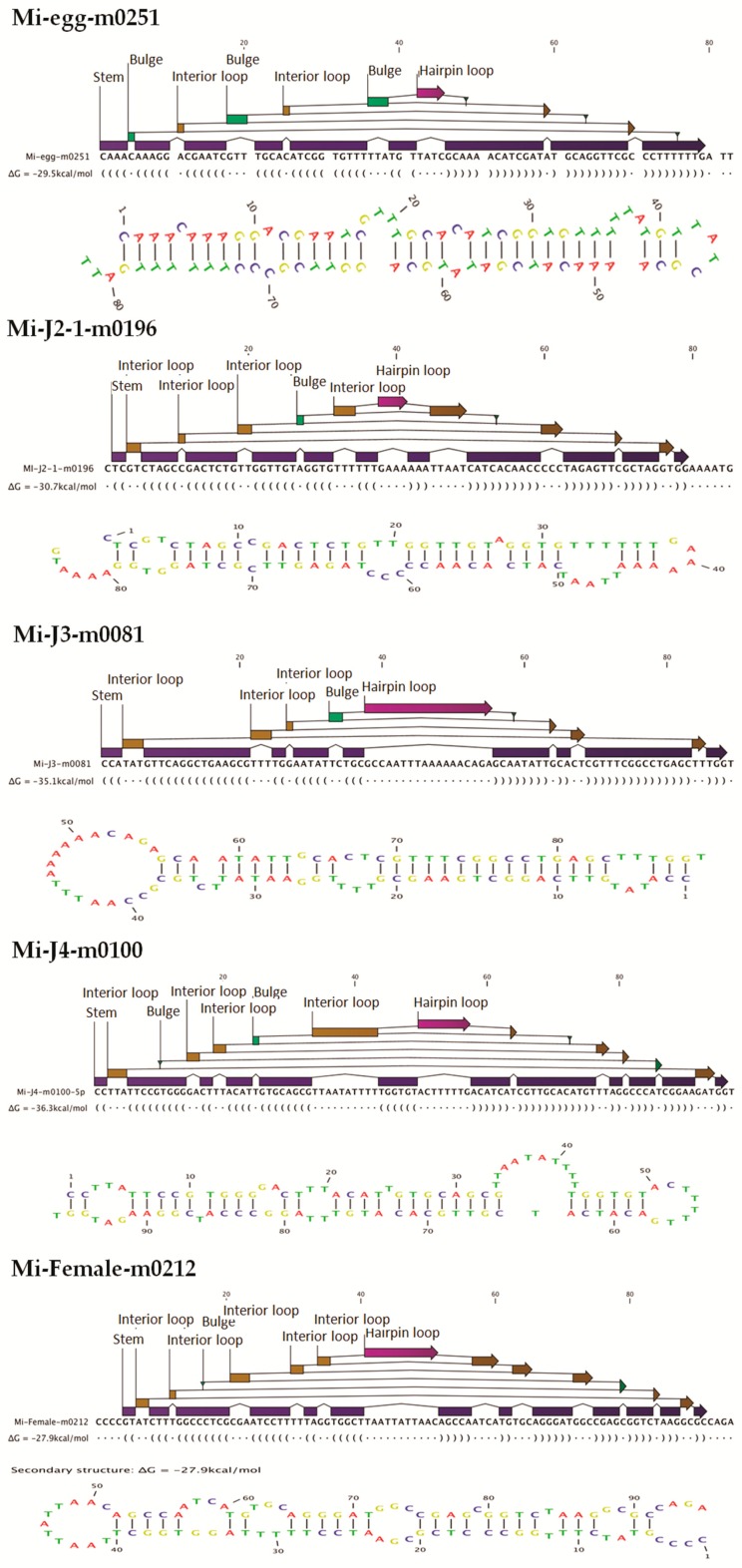
Pre-miRNA structure prediction of novel miRNAs from each stage of the *M. incognita* life cycle. Figures show structures, free energy values, and stem-loop structures for selected novel miRNAs.

Known and novel miRNA families were predicted using miRBase and MIREAP software, respectively, in comparison with the reference genome (ASM18041v1). We selected sequences with no more than two mismatches and a free gap around the seed region to be considered as a miRNA. Initially, the total number of annotated miRNA families identified were 9325, which were distributed across the egg, J2, J3, J4, and female stages (Table 2). These were considered known miRNAs. On removing duplicates at each stage, we obtained a total of 7627 known miRNA families across all the stages, which were further reduced to 3878 unique miRNA families. Similarly, MIREAP analysis of the unannotated sRNAs indicated 1407 novel miRNAs distributed across all the stages (Table 2 and Table S2). After eliminating duplicate reads we obtained a total of 778 unique novel miRNAs (Table S3). Following this, sequences with a read count above 10 (*R* > 10) from the unique miRNA reads were listed and re-assorted to combine miRNA subfamilies, which resulted in 2724 known and 383 novel miRNA families (Table 2) (Tables S4 and S5). Highly expressed known miRNA across all stages included miR-58, miR-1c, miR-124b, miR-71, miR-7062, miR-30e, miR-228, miR-100a, miR-6763, let-7, miR-72, miR-425, miR-7904, miR-3526, and miR-716b (Table 3). On studying novel miRNAs, the length of the pre-miRNAs in all the stages of the nematode life cycle was found to occur at a range of 60–109 nucleotides and we observed that the majority of the pre-miRNAs occurred at a range of 80–99 nucleotides (Figure S2). All the predicted novel miRNAs were confirmed with their characteristic stem-loop structures (Figure 4, Supplementary data: Novel miRNA secondary structures). Also, the minimal folding free energy index (MFEI) values were calculated (Table S6). We observed that 41.7% of the sequences (587 reads) had a negative MFEI value of over 0.85, while the remaining 58.2% of the sequences (820 reads) showed values less than 0.85. Highly expressed novel miRNAs at each developmental stage are shown in Table 4.

**Figure 5:**
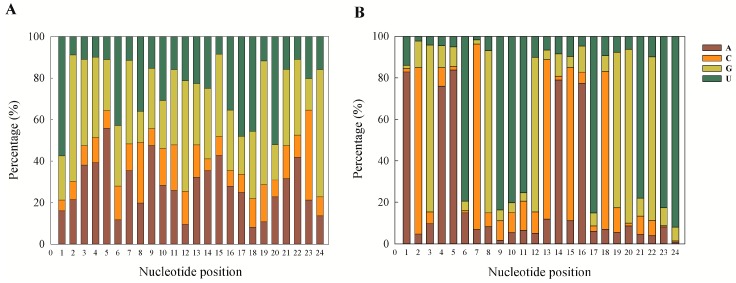
Nucleotide bias at each nucleotide position of known and novel miRNA of all developmental stages combined. (**A**) Known miRNA from annotation aligned to the miRNA precursor/mature miRNA in miRBase (allowing two mismatches and free gaps); (**B**) novel miRNAs predicted from the unannotated small RNAs using MIREAP software (Read ≥ 3).

Among novel miRNAs, the highly expressed families across the developmental stages of *M. incognita* are mentioned in Table 5. Studying the dominance of nucleotide bases at the first and ninth positions among the miRNAs shows that U and A were the preferred bases at these positions (Figure 5). Among the known miRNAs, the preferred first nucleotide base was U (57.47%), and at the ninth position A was found to be dominant (47.52%) (Figure 5A). In the case of novel miRNAs predicted using MIREAP software, the base A was found to be preferred at the first position (82.81%) and the ninth position was dominated by U (83.65%) (Figure 5B).

Comparison of miRNA families conserved in other nematodes was tabulated (Table 6). We found that among the compared nematodes, which contain pathogens and free-living nematodes, miRNA families miR-266, miR-2208, miR-2209, and miR-5593 were conserved only in *M. incoginta* and *C. elegans.* The miRNA families that were absent in *M. incognita* were miR-52, miR-59, miR-62, miR-237, miR-245, miR-258, miR-259, miR-354, miR-355, miR-356, miR-358, miR-786, miR-789, miR-791, miR-1829, miR-4922, and miR-5592. These miRNAs were also found to be absent in the other parasitic nematodes *Brugia malayi*, *Ascaris suum*, and *Haemonchus contortus.* However, they are present in several species of *Caenorhabditis* (Table 6).

Cluster analysis showed that 91 clusters at a threshold of 2000 bp and each cluster contained a minimum of one miRNA family and a maximum of six miRNA families (Table S7). Six clusters that have been previously reported in *M. incognita* in other studies were confirmed in our analysis. We identified 36 novel clusters located across the *M. incognita* genome that were constituted by novel miRNA families (Table 7). These clusters have not been reported in any other organism, including other nematodes. Further, we identified 45 clusters that contained previously identified miRNAs and novel miRNA families. Eight clusters (in contigs MiV1ctg164, MiV1ctg192, MiV1ctg237, MiV1ctg268, MiV1ctg342, MiV1ctg430, MiV1ctg516, and MiV1ctg78) that have already been reported in other organisms were also found (Table S7). Mapping of the miRNAs revealed that 2147 known and 464 novel miRNAs could be mapped to the reference genome (ASM18041v1a). These miRNAs were distributed among exons, introns, and intergenic regions but were absent in both untranslated regions (3′ and 5′ UTRs) (Table S8).

### 2.4. Stage-Specific Expression of M. incognita miRNA

**Figure 6:**
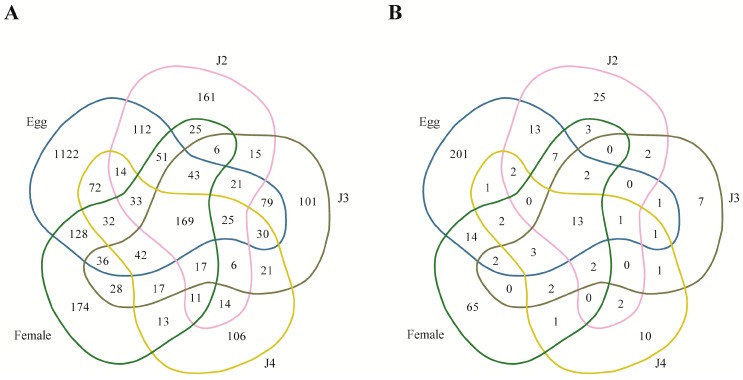
Distribution of miRNA expression across stages of the *M. incognita* life cycle. (**A**) Known miRNA (2724); and (**B**) novel miRNA (383).

Studying the stage-wise expression showed that among the known miRNAs, 169 families were commonly expressed across all stages (Table S9; Figure 6). Stage-specific known miRNAs were also observed: the egg stage had the maximum number of stage-specific miRNAs (1122 families), followed by the female stage, which showed 174 stage-specific miRNAs (Figure 6A) (Table S10). On studying the highly shared miRNAs between two stages, we found 128 miRNAs to be expressed both in the egg and female stages, followed by 112 miRNAs being expressed in the egg as well as the J2 stage. The least number of commonly expressed miRNAs among the stages were observed in J2, J3, and J4 stages (miR-1718, miR-3654, miR-622, miR-6883, miR-7631, and miR-7930) and, similarly, six miRNAs were commonly expressed across the J2, J3, and female stages (miR-1268, miR-1728, miR-199a, miR-3095, miR-4037, and miR-4534).

Among the novel miRNA, 13 families (MIN00015, MIN00018, MIN00053, MIN00068, MIN00075, MIN00079, MIN00113, MIN00131, MIN00284, MIN00308, MIN00321, MIN00333, and MIN00375) were commonly expressed in all stages (Figure 6B). The maximum number of stage-specific miRNA families was found in the egg stage, which was also the case in the known miRNAs. Stages J3 and J4 had the least number of specific novel miRNAs (Figure 6B). Similar to the known miRNAs, the highest number of shared miRNAs between two stages was found to be between the egg and female stages (14 miRNAs). Also, the second highest number of shared miRNAs was found to between the egg and J2 stages (13 miRNAs). This trend is similar for both known as well as novel miRNAs: a high number of miRNAs are shared between the egg and female stages in addition to the egg and J2 stages. The most highly expressed known miRNAs specific to each stage are miR-286, miR-2401, miR-8, miR-6736, and miR-17 in the egg, J2, J3, J4, and female stages, respectively. In the case of novel miRNA, MIN00001, MIN00016, MIN00005, MIN00022, and MIN00021 showed the highest expression in the egg, J2, J3, J4, and female stages, respectively (Table 8 and Table S10).

**Figure 7:**
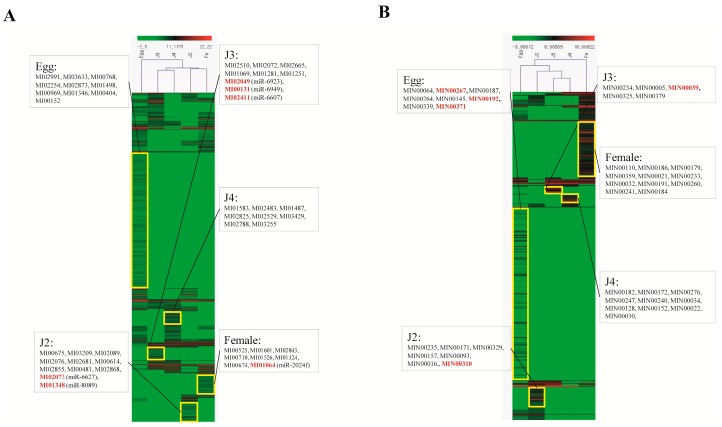
Heat map for the DESeq2-normalized expression profiles of known and novel miRNA families in five developmental stages. (**A**) Known miRNA; (**B**) novel miRNA. In both cases, sequences with *R* > 10 were selected for normalization. The heatmap of the normalized data (Tables S11 and S12) was drawn using MeV (v4.8.1) Pearson correlation applied for clustering. The TreeView tool was used to improve the figure clarity.

Expression patterns of known and novel miRNA were illustrated using heatmap and hierarchical clustering. In both known and novel miRNA expression, we found the egg stage to have the highest number of stage-specific miRNAs (Figure 7). In the case of known miRNAs, we observed clustering of the J3 and J4 as well as the J2 and female stages to form two subclades. The egg stage formed a different clade and was far from the expression profiles of the other stages (Figure 7A). Among novel miRNAs, primary clustering was also between the J3 and J4 stages. The female stage miRNA expression profile was the most distant compared to the other stages (Figure 7B).

### 2.5. In Vitro Confirmation of Specific miRNA Expression

**Figure 8:**
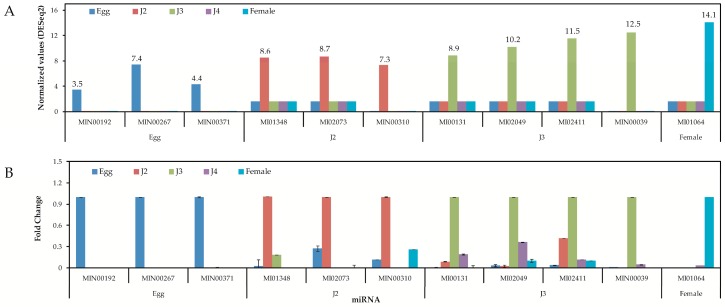
Validation of sequencing and real-time expression of stage-specific miRNAs. (**A**) Expression profiles of selected known and novel stage specific miRNA from high-throughput sequencing data. The normalized values of the stage-specific miRNAs are given at the top of the columns. Columns with DESeq2 values not mentioned had a normalized score of 0.1 to 1.6; (**B**) relative expression levels of known and novel miRNA studied using qPCR. The miRNA MI03018 (miR-58) was used as an internal standard.

High-throughput sequencing results of miRNA represented as read counts were normalized by DESeq2 normalization and normalized values were taken as a measure of miRNA expression level. Of the three commonly expressed miRNA candidates for internal standards, we observed similar expression patterns during qPCR studies (Figure S3). Upon experimental verification using quantitative PCR, we observed that all the chosen miRNAs were expressed in the nematode. Expression profiles of the 40 miRNAs were consistent when we compared expression with internal standards MI03018 and MI02743. On studying stage specificity, four of 10 miRNAs in the egg, three of nine in J2, all five in J3, and one of nine miRNAs in female were found to show specific expression (Figure S4). We were unable to detect any stage-specific miRNAs in the J4 stage from the stage-specific miRNAs selected from our sequencing data. During our experimental validation, few miRNAs were putatively known to be stage-specific; they also exhibited expression in other stages. However, their expression was highest in the stages at which they were characterized to be stage-specific by high-throughput sequencing data. A set of the selected stage-specific miRNA, their sequencing, and the qPCR results are shown in Figure 8.

## 3. Discussion

Reports on the sRNAs in *M. incognita* are currently emerging that indicate its importance as a plant parasitic nematode, and this report aims to study the stage-wise expression of miRNA across various stages of its development. This is the first summary of information on its sRNA across all stages and can serve as a potential resource for miRNA-mediated control studies on *M. incognita* in the future. Reports of miRNA-mediated regulation of *C. elegans* genes across developmental stages are available that can provide a model for other nematodes [13,19,20]. However, a comprehensive report on miRNAs from plant parasitic nematodes such as *M. incognita* can be helpful to identify and study the regulation of plant parasitism-related genes in nematodes.

Earlier studies in miRNA from *M. incognita* provide an excellent model to identify and study miRNAs of the RKN but are limited by the stages covered across the nematode life cycle [15,18]. In the present study, we sequenced small RNAs from all five stages of the *M. incognita* life cycle. During our analysis, preliminary mapping of known sequence reads showed a high percentage of mapping that was greatly reduced when unique sequences were mapped to the genome. This was also observed earlier and reported by Wang and coworkers, who explained genetic polymorphisms, the incompleteness of the genome, and sequencing errors as reasons for the phenomenon [15]. In our results, more than 95% of clean reads obtained from sequencing were longer than 18 nt. In general, the majority of the sRNAs range from 21 to 24 nt in size—information that is particularly helpful during initial predictions [17]. Also, an sRNA length of 23 nt was found to be dominant in unique sequence reads, which is consistent with earlier reports on *M. incognita* sRNAs [15]. Moreover, we observed that miRNAs and unannotated RNAs were the dominant classes of sRNAs, as also observed in a previous study involving sRNAs from the J2 stage of the nematode *M. incognita* [15].

A total of 9325 known microRNA families and 1407 novel families (read count > 3) were identified in our study. In both cases, the egg stage contributed to the highest amount of microRNAs (known—37.8%; novel—46.1%). Highly expressed known miRNA families include miR-58, miR-1c, miR-124b, miR-71, miR-7062, miR-30e, miR-228, miR-100a, miR-6763, and let-7. Although the exact function of these miRNAs in the *M. incognita* metabolism remains to be studied, we found reports on the probable function of these miRNAs in other species. The microRNA miR-1 has been known to target heat shock protein HSP60, causing glucose-mediated apoptosis in cardiac muscle cells [21]. miR-124 has been reported to be expressed in neuronal cells and target anti-neural function protein SCP1 and splicing repressor PTBP1 [22,23]. In the case of miR-30, the targets include an integrin ITGB3, ubiquitin-conjugating E2 enzyme UBC9, and tumor protein p53 [24,25]. The microRNA families miR-58 and miR-71 have been extensively reported in *Caenorhabditis elegans*, where miR-58 has been found to regulate the Transforming Growth Factor (TGF) pathway [26,27], and miR-71 functions in the control of aging in the model nematode [28]. Cluster analysis showed that the miRNA families, both known and novel, were localized to 91 clusters in the genome of *M. incognita*, where each cluster contained at least one gene of miRNA. We were able to confirm all clusters previously reported in *M. incognita* [15] except for the novel cluster consisting of NOVEL-1-1/NOVEL-39. Several of the clusters have also been found in other organisms, including *Drosophila*, mouse, other nematodes, and mammals (Table S7). MicroRNA families from *C. remanei* (miR-239b), *Brugia malayi* (miR-228), and *Ascaris suum* (miR-234) have hitherto not been reported in *M. incoginta* but reported in other nematodes (Table 6). We were able to map 2147 known and 464 novel miRNA to scaffold assembly set sequences (ASM18041v1a) of *M. incognita*. Mapping the microRNAs from several organisms showed that they are widely found to originate from the intergenic region or introns [26,29]. Our mapping data also show that our miRNAs were localized to introns and intergenic regions.

The number of known as well as novel miRNA in the present study were higher than the number of miRNAs predicted from *M. incognita* based from prior studies [15,30]. This is because earlier reports used no mismatches and gaps as conditions for miRNA prediction. However, in our study, we matched sequences with no more than two mismatches and free gaps around the seed region to be considered as candidate miRNAs. This is because, as explained earlier, the available genome data is a draft genome and may not be perfect. Our conditions helped to identify a high number of probable miRNA candidates that may have been lost by the stringent conditions used in the prior studies. MIREAP makes use of the sRNA secondary structure, dicer cleavage site, and the minimum free energy to predict novel miRNAs. Candidate novel miRNAs must satisfy two major criteria, namely the presence of a ∼22-nt miRNA sequence contained in one arm of the hairpin, which forms a fold-back precursor structure, and conservation of that ∼22-nt miRNA sequence as well as its secondary structure. Further, we also used the minimal free folding energy index (MFEI) values to confirm the pre-miRNA sequences [30]. In our studies, 41.7% of the novel miRNAs had negative MFEI values of over 0.85. The range of MFEI values for *M. incognita* miRNAs was reported to be 0.38 to 2.4 [18]. In our study, the average MFEI value was found to be 0.87, which suggests that the majority of the predicted novel miRNA are valid as miRNAs. Results of prior studies also showed miRNAs with MFEI values less than 0.85 [18]. The first nucleotide position of the known and novel mature miRNAs in the present study was found to be dominated by A and U. This is a ubiquitous phenomenon observed in miRNA studies [15,18,31,32]. This can also be taken as a putative marker for confirmation of sRNA sequences as miRNAs, and our results are also in accordance with previous reports.

Among the stage-specific miRNAs, known miRNAs specific to the egg stage include miR-286, which is highly specific to the embryo stages of *Drosophila melanogaster* [33] (Table 8). Other stage-specific miRNA families in the egg stage include miR-493, which is involved in the inhibition of liver metastasis in the mouse, and miR-27, which regulates cholesterol homeostasis and fatty acid metabolism [34,35]. Specific miRNA families from the J2 stage include miR-2401, miR-4451, and miR-2a, which have been reported to be synthesized by bovine animals in response to viral infection, occurring in normal and malignant human B cells, and involved in the repression of *Drosophila* pro-apoptotic factors, respectively [36,37,38]. In the J3 stage, specific miRNAs included miR-8 and miR-187, which are involved in the regulation of atrophin and the control of ovarian cancer, respectively [39,40]. Highly expressed specific miRNA families in the J4 stage included miR-6736, miR-2072-3, miR-7348, miR-2032a, and miR-2163. Specific miRNA families in the female stage are miR-17 and miR-3180, which are involved in cancers, and miR-133, which is reported to be expressed in muscle tissues [41,42,43].

Quantitative analysis of expression using PCR showed that the stage-specific miRNAs observed in the sequencing based studies were truly expressed in the nematode. We were able to confirm that these miRNAs were authentic and 14 of the 40 known and novel miRNAs in the egg, J2, J3, J4, and female exhibited stage-specific high expression patterns in both sequencing reads and quantitative PCR analyses. However, though the stage-specific miRNA candidate from the J4 stage was highly expressed in quantitative analyses, its expression was found to be high in the J3 stage when studied using PCR. Also, a few miRNAs that were shown to be stage-specific in digital expression studies were found to be expressed in other stages also, albeit in much lower quantities. Among the stage-specific known microRNAs taken for validation, MI01348 has been reported earlier in humans [44,45]. Other known stage-specific microRNAs were reported to be expressed in the mouse (MI02073), the chicken (MI00131), and chordate and cnidarian species (MI01064) [44,46,47,48]. However, the functions of these microRNAs in *M. incognita* remain to be elucidated and hitherto are only collected in an miRNA database—miRBase. In summary, through the present study, we have tried to amend the available data on *M. incognita* miRNAs to include miRNAs from all stages of development. This information can be very useful for any studies on the stage-specific control of the nematode or stage-specific functions in the future.

## 4. Materials and Methods

### 4.1. Sample Preparation

The nematode *M. incognita* was maintained in RKN-susceptible tomato plant roots (*Solanum lycopersicum* var. Rutgers) in a greenhouse at 25 °C. A fresh batch of plants was prepared for collection of *M. incognita* at various stages. Nematode samples were collected quickly at each developmental stage, i.e., egg, J2, J3, J4, and female, and snap frozen using liquid nitrogen, followed by temporary storage at −70 °C. Total RNA from each sample was extracted using CoreZol reagent (Corebio, Seoul, Korea) as per the manufacturer′s instructions.

### 4.2. Small RNA Library Construction

A flowchart depicting the flow of experiments carried out is explained in the abstract graphic. Isolation of small RNAs and library construction were performed as described by [49]. In brief, from the isolated total RNA, nucleotides of length 18–30 bp were obtained by gel separation and the purified short nucleotide RNA sequences were ligated with 3′ adapter 6–20 nt and 5′ adapter 12–25 nt at both ends, which was followed by purification according to the manufacturer’s protocol (T4 RNA Ligase, 200 U, 30 U/µL, Takara: D2050, Seoul, Korea). Ligated small RNAs were reverse transcribed with the complimentary sequence of 3′ adaptor (SuperScriptH III Reverse Transcriptase, Invitrogen: 18064-014, Carlsbad, CA, USA), and amplified. Finally, the PCR amplification products were sequenced at BGI (Beijing Genome Institute, Beijing, China) using Illumina HiSeq 2000 (Illumina Inc., San Diego, CA, USA) [50].

### 4.3. Annotation of M. incognita sRNAs

The software developed and provided by BGI was used to profile the data from high-throughput sequencing. After obtaining the FASTQ format files, the data were processed as per the following steps: (1) exclusion of low quality reads; (2) elimination of reads with 5′ primer contaminants; (3) elimination of reads without 3′ primer; (4) removal of reads without the insert tag; (5) removal of reads containing poly A and (6) elimination of reads shorter than 18 nt. The final clean reads were obtained and their length distribution was summarized. These clean read raw data are available at National Center for Biotechnology Information-Sequence Read Archive (NCBI-SRA) with accession numbers SRP076824, SRP077023, SRP077024, SRP077025, and SRP077026. In the present study, two versions of the reference genome were used, i.e., the Contig assembly sequence set (ASM18041v1) from NCBI was used for annotation and miRNA prediction, whereas scaffold assembly set sequences (ASM18041v1a) from the WormBase database (Available online: http://www.wormbase.org/) were used for miRNA mapping.

Clean reads of length ranging from 18 to 30 nt were analyzed to classify the small RNAs. First, the purified tags from the short clean reads were compared to the *M. incognita* genome sequence from NCBI (ASM18041v1). To find repeat associated RNA, small RNA tags were aligned to the reference genome using tag2repeat (BGI). The ribosomal RNA (rRNA), small nucleolar RNA (snoRNA), small nuclear RNA (snRNA), and transfer RNA (tRAN) were identified by Basic Local Alignment Search Tool (BLAST-NCBI) against the Genbank and Rfam databases. Small RNA tags were aligned to the reference genome, particularly the gene co-ordinates (exons and introns), to find the degraded fragments of mRNA by BGI-overlap software (Genome Institute, Beijing, China). To make every unique small RNA is mapped to only one annotation, the classification was followed by BGI small RNA pipeline (tag2annotation-BGI) such as rRNA, small cytoplasmic RNA (scRNA), small nucleolar RNA (snoRNA), small nuclear RNA (snRNA), tRNA, repeat sequences, and degradation mRNA, i.e., exons (sense and antisense) and introns (sense and antisense); the remaining reads were classified as unannotated small RNAs.

### 4.4. M. incognita miRNA Analysis

Known miRNAs were identified by matching the sRNAs to miRBase (v. 18) animal mature miRNA database using Tag2miRNA (BGI). Matched sequences with no more than two mismatches and two gaps around the seed region were considered as candidate conserved miRNAs (mismatches ≤ 2, and gaps ≤ 2 around the seed region). A small RNA around the location of an annotated pre-miRNA had at least 16 nt overlap, aligning to known miRNAs in miRBase. Also, they were aligned to the pre-miRNA and mature miRNA of all plants and animals in miRNAs, allowing two mismatches and free gaps. Any candidate small RNA sequence with more than three reads was considered a reliable representation of a miRNA molecule. Unannotated sRNAs that did not belong to any class of RNA but could be aligned to the reference genome were subjected to novel miRNA prediction using MIREAP software (Available online: http://sourceforge.net/projects/mireap) with default parameters. Candidate novel miRNAs satisfying two major criteria, namely presence of a ~22-nt miRNA sequence contained in one arm of the hairpin which when predicted forms a fold-back precursor structure and conservation of that ~22-nt miRNA sequence and its secondary structure were selected. The number of mature miRNAs with predicted hairpin was not less than five in the alignment result. Secondary structures of the miRNAs were confirmed for the presence of stem-loop structure. Pre-miRNA sequences from MIREAP results were used to calculate the minimal folding energy index (MFEI) [30].

The family distribution of the miRNAs and their nucleotide bias were obtained statistically to reduce any bias in the expression and coding of the miRNAs. For cluster analysis, miRNAs were grouped into clusters if they were located within a 2000 bp threshold in the genome during mapping [15]. Known and novel miRNAs with a read count >10 were mapped with reference to the *M. incognita* genome assembly database (ASM18041v1a; WormBase) to study their location in the genome. Stage-specific miRNAs (read count ≥ 3) were normalized using the DESeq2 package [51]. The DESeq2 normalized values of the miRNAs were taken as an estimation of the specific miRNA expression. For experimental validation we chose 40 stage-specific miRNAs combining all stages and three commonly expressed miRNAs as candidates for internal standards (Tables S13 and S14).

### 4.5. Experimental Validation of M. incognita miRNA Expression

Quantitative PCR analysis was used to experimentally validate the expression of miRNAs. Samples of the nematode *M. incognita* were taken at different developmental stages (egg, J2, J3, J4, and female) and used to isolate total RNA. Nematodes of each developmental stage were carefully collected and total RNA was extracted, followed by storage at −70 °C. Synthesis of cDNA from total RNA samples was carried out using Universal cDNA synthesis kit II (Exiqon, Woburn, MA, USA). For qPCR ExiLENT SYBR^®^ Green kit from Exiqon (Woburn, MA, USA) was used and qPCR was performed on ABI 7900HT Fast Real-Time PCR System (Applied Biosystems, Foster City, CA, USA) with S.D.S 2.2.2 software, as follows: 95 °C for 5 min, followed by 40 cycles of 95 °C for 10 s, 60 °C for 60 s with three independent replications containing three technical repeats each time. Negative control sets with no cDNA and no master mix were also tested. Three miRNAs, MI02950 (miR-71-5p), MI03018 (miR-58-3p), and MI02743 (miR-7904-3p), which consistently had analogous expression profiles at all stages of the nematode life cycle, were analyzed to be used as internal control for qPCR studies. The real-time qPCR studies were carried out as two independent experiments with at least three technical repeats. We calculated the fold changes in gene expression from the ΔΔ*C*_t_ values for all the selected miRNAs.

## 5. Conclusions

The RKN *M. incognita* is a major agricultural pest and environmental friendly solutions against use of harmful nematicides would be the goal in the future. Studying miRNA at each stage of development provides us with valuable resource for several aspects of study including, physiology and metabolism of the nematode at each stage, development of stage-specific markers, candidates for efficient control of the nematode and more. This study contributes to the existing database of miRNA from *M. incognita* and aims to serve as a useful source of information for future miRNA based studies.

## Figures and Tables

**Table 1:** Statistics of small RNAs obtained from high-throughput sequencing.

Type	Egg	%	J2	%	J3	%	J4	%	Female	%
Total reads	62,073,591	100	37,620,609	100	38,474,311	100	34,530,132	100	39,252,166	100
Low quality	248,004	0.40	159,876	0.42	403,270	1.05	234,574	0.68	203,453	0.52
High quality	61,825,587	99.60	37,460,733	99.58	38,071,041	98.95	34,295,558	99.32	39,048,713	99.48
In high quality reads *										
3′ Adapter null	36,544	0.06	27,388	0.07	76,270	0.20	29,037	0.08	32,300	0.08
Insert null	29,390	0.05	38,451	0.10	20,143	0.05	30,375	0.09	37,074	0.09
5′ Adapter contaminants	769,463	1.24	339,565	0.91	85,432	0.22	323,666	0.94	992,957	2.54
<18 bp	405,391	0.66	851,846	2.27	1,438,645	3.78	1,001,227	2.92	336,038	0.86
Poly-A	3226	0.01	331	0.00	440	0.00	251	0.00	480	0.00
Clean reads	60,581,573	97.99	36,203,152	96.64	36,450,111	95.74	32,911,002	95.96	37,649,864	96.42

* To proceed with annotation of sRNA the following have to be excluded from the sequencing results: (1) low-quality reads; (2) 5′ adapter contaminants; (3) reads without 3′ primer; (4) reads without the insert tag; (5) reads with poly A; and (6) reads shorter than 18 nt.

**Table 2:** Statistics of miRNA combining all developmental stages.

Known					Novel			
Clean Data			Reassortment		Clean Data		Reassortment	
Stages	miRNA Families (*R* ≥ 3 *)	Unique miRNA Families	*R* > 10 ^#^	Final Unique	miRNA Candidates (R ≥ 3)	Unique miRNA Candidates	*R* > 10	Final Unique
Egg	3529	2975	2069	2724	649	488	378	383
J2	1556	1221	806		199	131	106	
J3	1752	1091	735		88	55	59	
J4	1321	1035	713		108	63	53	
Female	1167	1305	825		363	250	178	
Total	9325	7627	5218		1407	987	774	
Unique		3878				778		

* *R* ≥ 3 read count greater than or equal to 3; ^#^ *R* > 10 read count greater than 10, reassortment-combining repeated subfamilies of miRNA and sequences with high sequence similarity.

**Table 3:** Highly expressed known miRNA families of *Meloidogyne incognita* (*M. incognita*).

miRNA Family	Known miRNA ID	DESeq2 Normalized Values					Sequence (5′ to 3′)	Length	G + C Content
		Egg	J2	J3	J4	Female			
miR-58	MI03018	22.22	18.65	16.24	17.50	20.41	TGAGATCAGTCCAGATTCGT	20	45
miR-1c	MI03171	21.33	19.73	16.15	13.96	17.46	TGGAATGTAAAGAAGTATGTAGA	23	30
miR-124b	MI02500	20.74	18.58	14.03	13.43	12.68	TAAGGCACGCGGTGAATGCTGA	22	55
miR-71	MI02950	20.52	20.71	19.54	18.75	20.06	TGAAAGACATGGGTAGTTGAGACG	24	46
miR-7062	MI03037	18.10	17.61	19.19	17.90	19.27	TGAGGACTGCTTGTGGAGTGCT	22	55
miR-30e	MI01618	11.70	11.11	16.21	17.52	19.57	CTTTGATCGGATGATTTGT	19	37
miR-228	MI00377	19.36	17.84	16.91	15.93	17.17	AATGGCACCAAATGAATTCACGG	23	43
miR-100a	MI00166	19.26	15.31	13.21	12.83	13.64	AACCCGTAGATCCGAACTAGTCTT	24	46
miR-6763	MI03365	15.08	14.97	17.59	17.82	19.24	TGGGGAGTTTGGCTGGGGCG	20	70
let-7	MI03059	12.43	10.67	15.71	17.97	19.02	TGAGGTAGTAGGTTGTATAGTT	22	36
miR-72	MI00819	18.48	16.32	12.11	12.84	13.18	AGGCAAGATGTTGGCATTGCTGA	23	48
miR-425	MI01048	16.80	16.97	18.41	8.75	17.29	ATCGGGGGTGTCGTAATCTTT	21	48
miR-7904	MI02743	18.27	15.73	15.40	17.01	15.71	TCAAAAATTCCGTTGCGTCGCA	22	45
miR-3526	MI03614	15.16	16.76	17.44	18.13	17.35	TTGAAGACTGAAGTGGAGA	19	42
miR-716b	MI01916	15.46	16.44	17.46	17.54	17.11	GCAGATCTTGGTGGTAGTAGCAAAT	25	44

Table shows highly expressed known miRNA across all stages. Read counts from the sequencing data were normalized to obtain DESeq2 values.

**Table 4:** Novel miRNA identification using MIREAP to distinguish pre miRNA among unannotated sRNA.

ID	Read Count	miRNA Precursor Sequence (5′ to 3′)	Seq Length	A%	T%	G%	C%	(A + T)%	(G + C)%	MFEI
Mi-egg-m0251	485,952	CAAACAAAGGACGAATCGTTTGCACATCGGTGTTTTTATGTTATCGCAAAACATCGATATGCAGGTTCGCCCTTTTTTGATT	82	26.8	34.1	19.5	19.5	61.0	39.0	−0.92
Mi-egg-m0191	12,935	TTGACAAAAAAACTTACGGACATATTTTTGCGGGAGGATGGAGAGGTATAGAGTCCGCAAAATCTGTCCGCAAGTCGCTGTCTGTCCG	88	28.4	25.0	27.3	19.3	53.4	46.6	−0.66
Mi-egg-m0462	8712	TGTCACCGTCCAAATTACATTCCTGTCCAAATCAATTTTGCCGGCCGAATTATATTTGGACAAGAATGTAATTTGGACGGTGACATT	87	28.7	32.2	18.4	20.7	60.9	39.1	−1.49
Mi-egg-m0003	8299	ATTGATCCGCACTTGTAGTGGTGTAAGCTTGTTTTGTAAAAGCAATTTTGTTGCGCCCAGGCAAATAAGCTCGCTTCTACAGGCGTGGGTCTTC	94	21.3	33.0	25.5	20.2	54.3	45.7	−0.91
Mi-egg-m0089	3922	TTTCCGTACTTGGCTCAAAGTTGGACAATTTATATGTTAATCGAAAGAGCTCGTTGATAAATTGTCCATTTTTGAACCAATTACGGAAAGT	91	30.8	35.2	18.7	15.4	65.9	34.1	−1.29
Mi-J2-1-m0196	7496	CTCGTCTAGCCGACTCTGTTGGTTGTAGGTGTTTTTTGAAAAAATTAATCATCACAACCCCCTAGAGTTCGCTAGGTGGAAAATG	85	25.9	31.8	22.4	20.0	57.6	42.4	−0.82
Mi-J2-1-m0123	5502	ATAGCCGATCGTTTGGGTTGATGATACGAACAATTGACCTACACCTTGCTGTGACAACATGTTCTTGATCGTTGACGCAACCACCTTGAGTAAC	94	26.6	28.7	21.3	23.4	55.3	44.7	−0.45
Mi-J2-1-m0195	1377	ACGCGCCGCCTGCTCCGGCATTTCTCTCGCTAGGGCCCTTGCGATAGATAGTTGGCTGTTTCGGTTATGGCAGTC	75	12.0	29.3	29.3	29.3	41.3	58.7	−0.54
Mi-J2-1-m0093	898	GATTGCGACCAGGCGTCGTCTCCGGCGGTTTCGAGTTAACAGTAAAAGCTGAAGCCGTAGGAGACGTCGTTAGGTTGCCTTCTC	84	20.2	25.0	31.0	23.8	45.2	54.8	−0.99
Mi-J2-1-m0041	888	CTTCGAGGCGGAATCCGGCTATCAATTCGCGCGGTTCCCCGTCCACAAGATTGCGAAGCAAAGTGAGTTTCTCGATGTCGGACCCTGAACTCGAGT	96	21.9	22.9	27.1	28.1	44.8	55.2	−0.57
Mi-J3-m0081	272	CCATATGTTCAGGCTGAAGCGTTTTGGAATATTCTGCGCCAATTTAAAAAACAGAGCAATATTGCACTCGTTTCGGCCTGAGCTTTGGT	89	25.8	31.5	22.5	20.2	57.3	42.7	−0.92
Mi-J3-m0062	182	TTTAATTAAAGAGCAATGAAGAAATATTTGACTAGAGTCTCAGAAAAATAATTTTGTTGAAAATTTTAAAGTCGGAGCTCTTTGAAAAGAT	91	41.8	33.0	17.6	7.7	74.7	25.3	−0.80
Mi-J3-m0040	110	TAGTTCTTGGATGGGTGTGCCTCTCCAGTCGTGTTTTTGGCCTAGTGCCACTATTTGGCTCATGACTAGATCCACACTCATCTAAGCACTT	91	17.6	35.2	22.0	25.3	52.7	47.3	−0.89
Mi-J3-m0036	100	TCCATGTTTTGACTGACCGTGTCTGTCCGTGTTTTGATGGCTCTTCAGGCAAAACGCGGACAGACACGGCCAGTCAAAACACGG	84	22.6	25.0	26.2	26.2	47.6	52.4	−1.29
Mi-J3-m0005	82	TCCACAATTTTTCCACAGCATGAACATCGGACATGACAAATATTGAAGTGTCCGATGTTCATGCTTAGGAATTATTGTGGAA	82	31.7	30.5	19.5	18.3	62.2	37.8	−1.58
Mi-J4-m0100	1385	CCTTATTCCGTGGGGACTTTACATTGTGCAGCGTTAATATTTTTGGTGTACTTTTTGACATCATCGTTGCACATGTTTAGGCCCATCGGAAGATGGT	97	19.6	38.1	23.7	18.6	57.7	42.3	−0.92
Mi-J4-m0006	704	ATTCTCGTCTAGCCGACTCTGTTGGTTGTAGGTGTTTTTAAAAATAATCATCACAACCCCCTAGAGTTCGCTAGGTGGAAAATG	84	26.2	32.1	21.4	20.2	58.3	41.7	−0.88
Mi-J4-m0048	475	TTAGTCGTTTACCCTGTAGTCCCGAGCCGTTTGAGAGAGACGTTACTTAAAACGTTGTTTGAAACCAAACGGAACTCGAGATGGGGAAGACGATTAGAAA	100	31.0	25.0	26.0	18.0	56.0	44.0	−0.83
Mi-J4-m0102	475	ATTTTTAGTCGTTTACCCTGTAGTCCCGAGCCGTTTGAGACTATAAAACGTTGTTTGAAACCAAACGGAACTCGAGATGGGGAAGACGATTGAAAA	96	31.3	27.1	24.0	17.7	58.3	41.7	−0.84
Mi-J4-m0079	158	GCAGGAGGACGGGGTCTGGCTTGGTTCTCATCTAGTATATACTTAGAAAACTGATGAGATCATACCAGATCACATTCGCTTGTTA	85	27.1	29.4	24.7	18.8	56.5	43.5	−0.82
Mi-Female-m0212	2151	CCCCGTATCTTTGGCCCTCGCGAATCCTTTTTAGGTGGCTTAATTATTAACAGCCAATCATGTGCAGGGATGGCCGAGCGGTCTAAGGCGCCAGA	95	21.1	26.3	26.3	26.3	47.4	52.6	−0.56
Mi-Female-m0286	1358	CATTTCGAGTTGAGATTTCTAATGAGCATTGTACGCCGCTAGTGAGACAGAAATTGTTTTATGTAAAATGCCCCTCTGAAATCTCAACTCGATTTTGT	98	27.6	34.7	19.4	18.4	62.2	37.8	−0.91
Mi-Female-m0221	1122	TTATGTGTATGTTGAGGTAGTAGGTTGTATAGTTAAAGAACAGTATCAGTCGGAGTAACTAACGCAGCCTGCTCACTCGGCAATCACATTC	91	28.6	29.7	24.2	17.6	58.2	41.8	−0.87
Mi-Female-m0142	457	ATTGAAATGAGCGGTCGTGTCCGAGTGGTTAAGGAGATTGACTCGAAATCAATTGGGCTCTGCCCGCACAGGTTCGAA	78	25.6	24.4	30.8	19.2	50.0	50.0	−0.65
MI-Female-m0140	300	GACAATCTTTGATTGATCGTCCTGACATTGTCATGCGGTTTTACGCTGACAAAGTTAGGGAGGTACACAAGGACTTGAA	79	27.8	29	25	17.7	57.0	43.04	−0.93

Id—miRNA identity, Seq length—sequence length, MFEI—minimal folding free energy index. Five highly expressed novel miRNA from each stage were taken and shown here to explain the processing of novel miRNAs.

**Table 5:** Highly expressed novel miRNA across all stages of the nematode life cycle.

miRNA ID	DESeq2 Normalized Values					Sequence (5′ to 3′)	Length	G + C Content (%)
	Egg	J2	J3	J4	Female			
MIN00284	11.40	13.60	12.17	11.85	9.34	TCACAACCCCCTAGAGTTCGCTAG	24	54
MIN00333	11.31	8.50	11.87	11.88	11.71	TGGGGACTTTACATTGTGCAGCG	23	52
MIN00079	9.35	9.59	11.16	11.01	8.31	AGGCTGAAGCGTTTTGGAATATT	23	39
MIN00018	10.30	10.20	9.90	9.36	11.61	AACTTACGGACATATTTTTGCG	22	36
MIN00308	10.33	8.12	6.54	8.36	6.54	TGAGATCATACCAGATCACAT	21	38
MIN00053	9.67	9.42	9.62	9.65	9.62	ACTTGTAGTGGTGTAAGCTTGTT	23	39
MIN00075	9.56	9.56	7.88	7.85	7.81	AGGCGTCGTCTCCGGCGGTTTC	22	68
MIN00375	8.71	8.28	7.25	7.25	9.81	TTTGGACAAGAATGTAATTTGGA	23	30
MIN00015	7.81	9.09	8.69	7.92	8.92	AACGCGGACAGACACGGCCAGT	22	64
MIN00321	7.23	8.40	8.03	6.53	6.91	TGCGGACATGTGGAGGACGAGC	22	64

Table shows highly expressed novel miRNA families across all the stages of the *M. incognita* life cycle. Read counts from sequencing data were compared and normalized to obtain the DESeq2 values.

**Table 6:** Comparison of conserved miRNA families among other nematode species.

miRNA Family	*Meloidogyne incognita* (This Study)	Free-Living Nematodes						Parasitic Nematodes		
		*C. brenneri*	*C. briggsae*	*C. elegans*	*C. remanei*	*Panagrellus redivivus*	*Pristionchus pacificus*	*Ascaris suum*	*Brugia malayi*	*Haemonchus contortus*
let-7	+	+	+	+	+	−	+	+	+	−
lin-4	+	+	+	+	+	−	−	+	+	−
miR-1	+	+	+	+	+	+	+	+	−	+
lsy-6	+	−	+	+	−	−	−	−	−	−
miR-2	+	+	+	+	+	−	−	+	+	+
miR-34	+	+	+	+	+	+	−	+	+	−
miR-35	+	−	+	+	−	−	−	−	−	−
miR-36	+	+	+	+	+	−	−	+	+	+
miR-39	+	+	+	+	+	−	−	−	−	−
miR-42	+	−	+	+	+	−	−	−	−	−
miR-43	+	+	+	+	+	−	−	−	−	−
miR-44	+	+	+	+	+	−	+	+	+	+
miR-46	+	+	+	+	+	+	+	+	+	+
miR-48	+	+	+	+	+	−	−	−	−	−
miR-49	+	+	+	+	+	−	−	−	−	−
miR-50	+	+	+	+	+	−	−	+	+	+
miR-51	+	+	+	+	+	−	−	−	−	−
miR-52	−	+	+	+	+	−	−	−	−	−
miR-54	+	+	+	+	−	−	−	−	−	−
miR-55	+	−	+	+	+	−	−	−	−	−
miR-56	+	−	−	+	+	−	−	−	−	−
miR-57	+	+	+	+	+	−	−	+	+	−
miR-58	+	+	+	+	+	−	−	−	−	−
miR-59	−	+	−	+	+	−	−	−	−	−
miR-60	+	+	+	+	+	−	−	−	−	+
miR-61	+	+	+	+	+	−	−	−	−	−
miR-62	−	+	+	+	+	−	−	−	−	−
miR-63	+	+	−	+	+	−	+	−	−	−
miR-64	+	+	−	+	−	−	−	−	−	−
miR-67	+	−	+	+	+	−	−	+	+	−
miR-70	+	−	+	+	−	−	−	−	−	−
miR-71	+	−	+	+	+	+	+	+	+	+
miR-72	+	+	+	+	+	+	+	+	+	+
miR-73	+	+	+	+	+	−	−	−	−	−
miR-74	+	+	+	+	+	−	−	−	−	−
miR-75	+	+	+	+	+	−	−	−	−	−
miR-76	+	−	+	+	+	−	−	−	−	−
miR-77	+	+	+	+	+	−	−	−	−	−
miR-9	+	+	+	+	+	+	+	+	+	+
miR-80	+	+	+	+	+	−	−	−	−	−
miR-81	+	+	+	+	+	−	−	+	−	−
miR-83	+	+	+	+	+	−	−	+	−	+
miR-84	+	−	+	+	+	−	+	+	+	−
miR-85	+	+	+	+	+	−	−	−	−	−
miR-86	+	+	+	+	+	+	+	+	+	+
miR-87	+	+	+	+	+	+	+	+	+	+
miR-90	+	+	+	+	+	−	−	−	−	−
miR-124	+	+	+	+	+	+	+	+	+	+
miR-228	+	+	+	+	+	−	−	−	−	−
miR-230	+	−	+	+	+	−	−	−	−	−
miR-231	+	+	+	+	+	−	−	−	−	−
miR-232	+	+	+	+	+	−	−	−	−	−
miR-233	+	+	+	+	+	−	−	−	−	−
miR-234	+	+	+	+	−	+	+	+	+	+
miR-235	+	−	+	+	+	−	−	−	−	−
miR-236	+	+	+	+	+	+	−	+	+	+
miR-237	−	−	+	+	+	−	−	−	−	−
miR-238	+	−	+	+	+	−	−	−	−	−
miR-239	+	+	+	+	+	−	+	+	+	−
miR-240	+	−	+	+	−	−	−	−	−	−
miR-241	+	+	+	+	+	−	−	−	−	−
miR-242	+	−	+	+	+	−	−	−	−	−
miR-244	+	+	+	+	+	−	−	−	−	−
miR-245	−	+	+	+	+	−	−	−	−	−
miR-246	+	+	+	+	+	−	−	−	−	−
miR-247	+	−	−	+	+	−	−	−	−	−
miR-248	+	−	+	+	+	−	−	−	−	−
miR-249	+	−	+	+	+	−	−	−	−	−
miR-250	+	+	+	+	+	−	−	−	−	−
miR-251	+	+	+	+	+	−	−	−	−	−
miR-252	+	−	+	+	+	+	+	+	+	+
miR-253	+	−	+	+	+	−	−	−	−	−
miR-254	+	+	+	+	+	−	−	−	−	−
miR-255	+	+	+	+	+	−	−	−	−	−
miR-258	−	−	−	+	−	−	−	−	−	−
miR-259	−	−	+	+	+	−	−	−	−	−
miR-266	+	−	−	+	−	−	−	−	−	−
miR-268	+	−	+	+	−	−	−	−	+	−
miR-353	+	−	+	+	−	−	−	−	−	−
miR-354	−	−	+	+	−	−	−	−	−	−
miR-355	−	+	+	+	+	−	−	−	−	−
miR-356	−	−	+	+	+	−	−	−	−	−
miR-357	+	+	+	+	+	−	−	−	−	−
miR-358	−	−	+	+	−	−	−	−	−	−
miR-359	+	−	+	+	−	−	−	−	−	−
miR-360	+	+	+	+	+	−	−	−	−	−
miR-392	+	−	+	+	−	−	−	−	−	−
miR-785	+	−	+	+	+	−	−	−	−	−
miR-786	−	−	+	+	+	−	−	−	−	−
miR-787	+	−	+	+	−	−	−	−	−	−
miR-788	+	−	−	+	+	−	−	−	−	−
miR-789	−	−	+	+	−	−	−	−	−	−
miR-790	+	+	+	+	+	−	−	−	−	−
miR-791	−	−	+	+	−	−	−	−	−	−
miR-1822	+	+	+	+	+	−	−	−	−	−
miR-1829	−	−	−	+	−	−	−	−	−	−
miR-2208	+	−	−	+	−	−	−	−	−	−
miR-2209	+	−	−	+	−	−	−	−	−	−
miR-2214	+	−	+	+	−	−	−	−	−	−
miR-4922	−	−	−	+	−	−	−	−	−	−
miR-5592	−	−	−	+	−	−	−	−	−	−
miR-5593	+	−	−	+	−	−	−	−	−	−

*C.—Caenorhabdits*. The information was tabulated from results of the BGI (Beijing Genome Institute, Beijing, China) sequencing report. The data for comparison for miRNA families were obtained from miRNA database miRBase version 18.

**Table 7:** Novel miRNA clusters of *M. incognita* found in our study.

Contig	Start	End	Strand	Cluster
MiV1ctg1	228,236	228,259	+	MIN0559
	228,430	228,451	−	MIN0402
MiV1ctg103	108,004	108,026	−	MIN0712
	108,057	108,078	+	MIN0565
MiV1ctg1058	19,249	19,270	+	MIN0362
	19,968	19,989	+	MIN0509
MiV1ctg1143	4236	4257	+	MIN0626
	4348	4370	+	MIN0676
MiV1ctg122	59,164	59,186	+	MIN0138
	60,889	60,909	−	MIN0010
MiV1ctg13	112,247	112,270	−	MIN0353
	112,331	112,353	−	MIN0180
MiV1ctg151	81,937	81,960	−	MIN0158
	83,472	83,493	+	MIN0419
MiV1ctg155	69,676	69,699	+	MIN0455
	69,826	69,849	+	MIN0352
MiV1ctg162	44,780	44,801	−	MIN0719
	44,832	44,854	+	MIN0199
MiV1ctg172	64,117	64,138	−	MIN0030
	64,842	64,863	+	MIN0040
MiV1ctg181	62,265	62,286	−	MIN0552
MiV1ctg2	111,341	111,363	−	MIN0017
	111,800	111,822	+	MIN0092
MiV1ctg209	24,055	24,076	+	MIN0277
	24,898	24,921	−	MIN0517
MiV1ctg2099	2475	2497	−	MIN0509
	3234	3255	−	MIN0362
MiV1ctg220	66,966	66,988	+	MIN0591
MiV1ctg2865	1936	1958	+	MIN0631
	1989	2011	−	MIN0630
	1991	2013	+	MIN0020
MiV1ctg310	45,466	45,593	−	MIN0751
MiV1ctg346	30,081	30,101	+	MIN0007
	31,742	31,765	+	MIN0472
MiV1ctg384	43,585	43,606	−	MIN0040
	44,310	44,331	+	MIN0030
MiV1ctg387	53,738	53,760	+	MIN0017
	53,856	53,878	−	MIN0092
MiV1ctg394	26,272	26,294	+	MIN0636
	26,295	26,318	−	MIN0480
	26,361	26,383	−	MIN0319
	28,325	28,347	−	MIN0665
MiV1ctg42	107,915	107,937	−	MIN0188
	108,677	108,700	−	MIN0198
MiV1ctg47	170,763	170,784	−	MIN0682
	171,345	171,365	−	MIN0358
MiV1ctg547	11,747	11,769	−	MIN0398
	12,367	12,389	+	MIN0114
MiV1ctg554	34,722	34,743	+	MIN0058
	35,933	35,954	−	MIN0645
MiV1ctg614	19,086	19,108	+	MIN0770
	19,138	19,159	−	MIN0237
MiV1ctg638	37,469	37,618	+	MIN0720
MiV1ctg655	32,120	32,142	+	MIN0570
	32,904	32,924	+	MIN0155
MiV1ctg687	33,204	33,226	−	MIN0638
	33,418	33,440	−	MIN0086
MiV1ctg7	176,234	176,254	+	MIN0227
	177,333	177,354	+	MIN0567
MiV1ctg726	22,611	22,634	−	MIN0351
	24,144	24,166	−	MIN0180
MiV1ctg75	109,253	109,276	+	MIN0455
	109,403	109,426	+	MIN0352
MiV1ctg781	16,033	16,052	+	MIN0173
MiV1ctg876	10,111	10,133	+	MIN0501
	11,791	11,814	+	MIN0634
MiV1ctg889	12,209	12,232	−	MIN0116
MiV1ctg9	133,529	133,550	+	MIN0594
	133,572	133,593	−	MIN0672
	134,249	134,271	−	MIN0371

This table represents 36 novel clusters among the 91 clusters located across the *M. incognita* genome identified in the present study.

**Table 8:** Highly expressed stage-specific miRNAs of *M. incognita*.

miRNA	Stage	miRNA	Sequences (5′ to 3′)	Read Count
Known miRNA	Egg	miR-286	TGACTAGACAAACAACTCGTGT	137,573
	J2	miR-2401	AGAGTTTGACTAGGGCGG	122,762
	J3	miR-8	TAATAATGTAGGTAATGGAAGTCG	140,213
	J4	miR-6736	CTGGGGCGGCACATCTGTGGT	107,793
	Female	miR-17	ACTAGCAGTGAGGACTGCTTGTGG	107,167
Novel miRNA	Egg	MIN00001	AAAAACAGCTGAGTACTTGTCG	80
	J2	MIN00016	AACGGAACTCGAGATGGGGAAG	116
	J3	MIN00005	AAAATTTTAAAGTCGGAGCTCT	131
	J4	MIN00022	AAGGAAATTGGATGCCGGCATT	12
	Female	MIN00021	AAGCTTCCCAGTGGCGGAGTCG	244

## Supplementary Materials

Supplementary materials can be found at www.mdpi.com/1422-0067/17/10/1758/s1.

## Abbreviations

miRNA	MicroRNA
RKN	Root-knot nematode
PPN	Plant parasitic nematode
sRNA	Small RNA
RNAi	RNA interference
pre-miRNA	precursor miRNA
nt	nucleotide

## References

[1] Mitkowski N., Abawi G. (2003). Root-knot nematodes. Plant Health Instr..

[2] Moens M., Perry R.N., Starr J.L. (2009). Meloidogyne Species—A Diverse Group of Novel and Important Plant Parasites.

[3] Chitwood D.J., Plimmer J.R. (2003). Nematicides. Encyclopedia Agrochemical.

[4] Noling J. (2011). Movement and Toxicity of Nematicides in the Plant Root Zone.

[5] Bartel D.P. (2004). Micrornas: Genomics, biogenesis, mechanism, and function. Cell.

[6] Kim V.N. (2005). Microrna biogenesis: Coordinated cropping and dicing. Nat. Rev. Mol. Cell Biol..

[7] Huang G., Allen R., Davis E.L., Baum T.J., Hussey R.S. (2006). Engineering broad root-knot resistance in transgenic plants by RNAi silencing of a conserved and essential root-knot nematode parasitism gene. Proc. Natl. Acad. Sci. USA.

[8] Yadav B.C., Veluthambi K., Subramaniam K. (2006). Host-generated double stranded RNA induces RNAi in plant-parasitic nematodes and protects the host from infection. Mol. Biochem. Parasitol..

[9] Li J., Todd T.C., Lee J., Trick H.N. (2011). Biotechnological application of functional genomics towards plant-parasitic nematode control. Plant Biotechnol. J..

[10] Ajjappala H., Chung H.Y., Sim J.S., Choi I., Hahn B.S. (2015). Disruption of prefoldin-2 protein synthesis in root-knot nematodes via host-mediated gene silencing efficiently reduces nematode numbers and thus protects plants. Planta.

[11] Ameres S.L., Zamore P.D. (2013). Diversifying microRNA sequence and function. Nat. Rev. Mol. Cell Biol..

[12] Ha M., Kim V.N. (2014). Regulation of microRNA biogenesis. Nat. Rev. Mol. Cell Biol..

[13] Ambros V., Lee R.C., Lavanway A., Williams P.T., Jewell D. (2003). MicroRNAs and other tiny endogenous RNAs in *C. elegans*. Curr. Biol..

[14] Danchin E.G., Arguel M.J., Campan-Fournier A., Perfus-Barbeoch L., Magliano M., Rosso M.N., Da Rocha M., Da Silva C., Nottet N., Labadie K. (2013). Identification of novel target genes for safer and more specific control of root-knot nematodes from a pan-genome mining. PLoS Pathog..

[15] Wang Y., Mao Z., Yan J., Cheng X., Liu F., Xiao L., Dai L., Luo F., Xie B. (2015). Identification of microRNAs in *Meloidogyne incognita* using deep sequencing. PLoS ONE.

[16] Zhao W., Li Z., Fan J., Hu C., Yang R., Qi X., Chen H., Zhao F., Wang S. (2015). Identification of jasmonic acid-associated microRNAs and characterization of the regulatory roles of the miR319/TCP4 module under root-knot nematode stress in tomato. J. Exp. Bot..

[17] Abad P., Gouzy J., Aury J.M., Castagnone-Sereno P., Danchin E.G., Deleury E., Perfus-Barbeoch L., Anthouard V., Artiguenave F., Blok V.C. (2008). Genome sequence of the metazoan plant-parasitic nematode *Meloidogyne incognita*. Nat. Biotechnol..

[18] Zhang Y., Wang Y., Xie F., Li C., Zhang B., Nichols R.L., Pan X. (2016). Identification and characterization of microRNAs in the plant parasitic root-knot nematode *Meloidogyne incognita* using deep sequencing. Funct. Integr. Genom..

[19] Lall S., Grun D., Krek A., Chen K., Wang Y.L., Dewey C.N., Sood P., Colombo T., Bray N., Macmenamin P. (2006). A genome-wide map of conserved microRNA targets in *C. elegans*. Curr. Biol..

[20] Kato M., de Lencastre A., Pincus Z., Slack F.J. (2009). Dynamic expression of small non-coding rnas, including novel micrornas and piRNAs/21U-RNAs, during *Caenorhabditis elegans* development. Genome Biol..

[21] Shan Z.X., Lin Q.X., Deng C.Y., Zhu J.N., Mai L.P., Liu J.L., Fu Y.H., Liu X.Y., Li Y.X., Zhang Y.Y. (2010). miR-1/miR-206 regulate Hsp60 expression contributing to glucose-mediated apoptosis in cardiomyocytes. FEBS Lett..

[22] Makeyev E.V., Zhang J., Carrasco M.A., Maniatis T. (2007). The microRNA miR-124 promotes neuronal differentiation by triggering brain-specific alternative pre-mRNA splicing. Mol. Cell.

[23] Visvanathan J., Lee S., Lee B., Lee J.W., Lee S.K. (2007). The microRNA miR-124 antagonizes the anti-neural REST/SCP1 pathway during embryonic CNS development. Genes Dev..

[24] Li J., Donath S., Li Y., Qin D., Prabhakar B.S., Li P. (2010). miR-30 regulates mitochondrial fission through targeting p53 and the dynamin-related protein-1 pathway. PLoS Genet..

[25] Yu F., Deng H., Yao H., Liu Q., Su F., Song E. (2010). miR-30 reduction maintains self-renewal and inhibits apoptosis in breast tumor-initiating cells. Oncogene.

[26] Lee R.C., Ambros V. (2001). An extensive class of small RNAs in *Caenorhabditis elegans*. Science.

[27] De Lucas M.P., Saez A.G., Lozano E. (2015). miR-58 family and TGF-β pathways regulate each other in caenorhabditis elegans. Nucleic Acids Res..

[28] Boulias K., Horvitz H.R. (2012). The *C. elegans* microRNA miR-71 acts in neurons to promote germline-mediated longevity through regulation of DAF-16/FOXO. Cell Metab..

[29] Rodriguez A., Griffiths-Jones S., Ashurst J.L., Bradley A. (2004). Identification of mammalian microRNA host genes and transcription units. Genome Res..

[30] Zhang B.H., Pan X.P., Cox S.B., Cobb G.P., Anderson T.A. (2006). Evidence that miRNAs are different from other RNAs. Cell. Mol. Life Sci..

[31] Huang Q.X., Cheng X.Y., Mao Z.C., Wang Y.S., Zhao L.L., Yan X., Ferris V.R., Xu R.M., Xie B.Y. (2010). MicroRNA discovery and analysis of pinewood nematode *Bursaphelenchus xylophilus* by deep sequencing. PLoS ONE.

[32] Kaufman E.J., Miska E.A. (2010). The microRNAs of *Caenorhabditis elegans*. Semin. Cell Dev. Biol..

[33] Aravin A.A., Lagos-Quintana M., Yalcin A., Zavolan M., Marks D., Snyder B., Gaasterland T., Meyer J., Tuschl T. (2003). The small RNA profile during drosophila melanogaster development. Dev. Cell.

[34] Fernández-Hernando C., Suárez Y., Rayner K.J., Moore K.J. (2011). MicroRNAs in lipid metabolism. Curr. Opin. Lipidol..

[35] Okamoto K., Ishiguro T., Midorikawa Y., Ohata H., Izumiya M., Tsuchiya N., Sato A., Sakai H., Nakagama H. (2012). miR-493 induction during carcinogenesis blocks metastatic settlement of colon cancer cells in liver. EMBO J..

[36] Glazov E.A., Kongsuwan K., Assavalapsakul W., Horwood P.F., Mitter N., Mahony T.J. (2009). Repertoire of bovine miRNA and miRNA-like small regulatory RNAs expressed upon viral infection. PLoS ONE.

[37] Jima D.D., Zhang J., Jacobs C., Richards K.L., Dunphy C.H., Choi W.W., Au W.Y., Srivastava G., Czader M.B., Rizzieri D.A. (2010). Deep sequencing of the small RNA transcriptome of normal and malignant human B cells identifies hundreds of novel microRNAs. Blood.

[38] Leaman D., Chen P.Y., Fak J., Yalcin A., Pearce M., Unnerstall U., Marks D.S., Sander C., Tuschl T., Gaul U. (2005). Antisense-mediated depletion reveals essential and specific functions of microRNAs in drosophila development. Cell.

[39] Lagos-Quintana M., Rauhut R., Lendeckel W., Tuschl T. (2001). Identification of novel genes coding for small expressed RNAs. Science.

[40] Chao A., Lin C.Y., Lee Y.S., Tsai C.L., Wei P.C., Hsueh S., Wu T.I., Tsai C.N., Wang C.J., Chao A.S. (2012). Regulation of ovarian cancer progression by microRNA-187 through targeting disabled homolog-2. Oncogene.

[41] Ivey K.N., Muth A., Arnold J., King F.W., Yeh R.F., Fish J.E., Hsiao E.C., Schwartz R.J., Conklin B.R., Bernstein H.S. (2008). MicroRNA regulation of cell lineages in mouse and human embryonic stem cells. Cell Stem Cell.

[42] Mendell J.T. (2008). miRiad roles for the miR-17-92 cluster in development and disease. Cell.

[43] Persson H., Kvist A., Rego N., Staaf J., Vallon-Christersson J., Luts L., Loman N., Jonsson G., Naya H., Hoglund M. (2011). Identification of new microRNAs in paired normal and tumor breast tissue suggests a dual role for the ERBB2/Her2 gene. Cancer Res..

[44] Ladewig E., Okamura K., Flynt A.S., Westholm J.O., Lai E.C. (2012). Discovery of hundreds of mirtrons in mouse and human small RNA data. Genome Res..

[45] Wang H.J., Zhang P.J., Chen W.J., Jie D., Dan F., Jia Y.H., Xie L.X. (2013). Characterization and identification of novel serum microRNAs in sepsis patients with different outcomes. Shock.

[46] Shao P., Liao J.Y., Guan D.G., Yang J.H., Zheng L.L., Jing Q., Zhou H., Qu L.H. (2012). Drastic expression change of transposon-derived pirna-like RNAs and microRNAs in early stages of chicken embryos implies a role in gastrulation. RNA Biol..

[47] Grimson A., Srivastava M., Fahey B., Woodcroft B.J., Chiang H.R., King N., Degnan B.M., Rokhsar D.S., Bartel D.P. (2008). Early origins and evolution of microRNAs and Piwi-interacting RNAs in animals. Nature.

[48] Moran Y., Fredman D., Praher D., Li X.Z., Wee L.M., Rentzsch F., Zamore P.D., Technau U., Seitz H. (2014). Cnidarian microRNAs frequently regulate targets by cleavage. Genome Res..

[49] Hafner M., Landgraf P., Ludwig J., Rice A., Ojo T., Lin C., Holoch D., Lim C., Tuschl T. (2008). Identification of microRNAs and other small regulatory RNAs using cDNA library sequencing. Methods.

[50] Chen Q., Lu L., Hua H., Zhou F., Lu L., Lin Y. (2012). Characterization and comparative analysis of small RNAs in three small RNA libraries of the brown planthopper (*Nilaparvata lugens*). PLoS ONE.

[51] Love M.I., Huber W., Anders S. (2014). Moderated estimation of fold change and dispersion for RNA-seq data with DESeq2. Genome Biol..

