# Supplementary material for: Stage-Wise Identification and Analysis of miRNA from Root-Knot Nematode Meloidogyne incognita
Source: Int J Mol Sci. 2016 Oct 21;17(10):1758. doi: 10.3390/ijms17101758 (PMC5085782; doi:10.3390/ijms17101758)
Supplement: Supplementary file 1 [file ijms-17-01758-s001.zip › 12. ijms-150713 supplementary materials/ijms-150713 suppl. .docx]

Supplementary Materials: Stage-Wise Identification and Analysis of miRNA from Root-Knot Nematode *Meloidogyne incognita*

Parthiban Subramanian, In-Chan Choi, Vimalraj Mani, Junhyung Park,
Sathiyamoorthy Subramaniyam, Kang-Hyun Choi, Joon-Soo Sim, Chang-Muk Lee,
Ja Choon Koo and Bum-Soo Hahn

**Table S1.** Preliminary mapping report of sRNA to *Meloidogyne incognita (M. incoginta)* genome.

| **Stage** | **Total** | | | **Unique** | | |
| --- | --- | --- | --- | --- | --- | --- |
|  | **Clean** | **Mapping** | **%** | **Clean** | **Mapping** | **%** |
| Egg | 60,581,573 | 49,155,332 | 81.14 | 4,314,279 | 2,244,367 | 52.02 |
| J2 | 36,203,152 | 30,515,292 | 84.29 | 1,414,282 | 565,374 | 39.98 |
| J3 | 36,450,111 | 17,243,392 | 47.31 | 1,759,861 | 365,221 | 20.75 |
| J4 | 32,911,002 | 14,028,763 | 42.63 | 1,458,207 | 392,928 | 26.95 |
| Female | 37,649,864 | 31,110,353 | 82.63 | 1,645,498 | 639,653 | 38.87 |

**Table S2.** Novel miRNAs identified from unannotated sRNAs using MIREAP software (BGI).

**Table S3.** Unique novel miRNA of *M. incoginta* from all stages with *R* > 3.

**Table S4.** Known miRNA of *M. incognita* from all stages with *R* > 10.

**Table S5.** Novel miRNA of *M. incoginta* from all stages with *R* > 10.

**Table S6.** Statistics and MFEI values of novel miRNA from *M. incognita.*

**Table S7.** Statistics of miRNA clusters of *M. incognita.*

| **Contig** | **Start** | **End** | **Strand** | **Cluster** | **Organism** |
| --- | --- | --- | --- | --- | --- |
| MiV1ctg0 | 275,545 | 275,565 | + | rno-miR-217-5p | *Rattus norvegicus* |
|  | 277,126 | 277,148 | − | MIN0477 | *Meloidogyne incognita* (this study) |
| MiV1ctg1 | 228,236 | 228,259 | + | MIN0559 | *Meloidogyne incognita* (this study) |
|  | 228,430 | 228,451 | − | MIN0402 | *Meloidogyne incognita* (this study) |
| MiV1ctg10 | 12,814 | 12,835 | + | MIN0668 | *Meloidogyne incognita* (this study) |
|  | 14,512 | 14,534 | + | MIN0133 | *Meloidogyne incognita* (this study) |
|  | 14,561 | 14,582 | + | dvi-miR-310/ | *Drosophila virilis* |
|  |  |  |  | bbe-miR-2059-5p | *Branchiostoma belcheri* |
|  | 14,559 | 14,585 | + | prd-miR-235-3p/ | *Panagrellus redivivus* |
|  |  |  |  | asu-miR-92-3p | *Ascaris suum* |
| MiV1ctg103 | 108,004 | 108,026 | − | MIN0712 | *Meloidogyne incognita* (this study) |
|  | 108,057 | 108,078 | + | MIN0565 | *Meloidogyne incognita* (this study) |
| MiV1ctg1058 | 19,249 | 19,270 | + | MIN0362 | *Meloidogyne incognita* (this study) |
|  | 19,968 | 19,989 | + | MIN0509 | *Meloidogyne incognita* (this study) |
| MiV1ctg1075 | 9140 | 9,161 | + | cfa-miR-329a | *Canis familiaris* |
|  | 9386 | 9,407 | + | MIN0177 | *Meloidogyne incognita* (this study) |
|  | 10,158 | 10,179 | − | MIN0095 | *Meloidogyne incognita* (this study) |
|  | 12,091 | 12,113 | + | pma-miR-190b-5p | *Petromyzon marinus* |
|  | 12,253 | 12,274 | + | MIN0656 | *Meloidogyne incognita* (this study) |
| MiV1ctg1092 | 13,269 | 13,290 | + | ppy-miR-2278 | *Pongo pygmaeus* |
|  | 14,315 | 14,336 | − | MIN0065 | *Meloidogyne incognita* (this study) |
| MiV1ctg1143 | 4236 | 4,257 | + | MIN0626 | *Meloidogyne incognita* (this study) |
|  | 4348 | 4,370 | + | MIN0676 | *Meloidogyne incognita* (this study) |
| MiV1ctg120 | 89,001 | 89,023 | + | MIN0473 | *Meloidogyne incognita* (this study) |
|  | 89,729 | 89,753 | − | aca-miR-301a-5p | *Anolis carolinensis* |
| MiV1ctg122 | 59,164 | 59,186 | + | MIN0138 | *Meloidogyne incognita* (this study) |
|  | 60,889 | 60,909 | − | MIN0010 | *Meloidogyne incognita* (this study) |
| MiV1ctg13 | 112,247 | 112,270 | − | MIN0353 | *Meloidogyne incognita* (this study) |
|  | 112,331 | 112,353 | − | MIN0180 | *Meloidogyne incognita* (this study) |
| MiV1ctg136 | 57,440 | 57,460 | + | ptr-miR-4428 | *Pan troglodytes* |
|  | 57,544 | 57,564 | − | MIN0699 | *Meloidogyne incognita* (this study) |
| MiV1ctg1479 | 13,885 | 13,909 | − | cel-miR-85-3p | *Caenorhabditis elegans* |
|  | 14,764 | 15,695 | − | eca-miR-539 | *Equus caballus* |
|  | 15,159 | 15,181 | + | MIN0135 | *Meloidogyne incognita* (this study) |
| MiV1ctg149 | 41,872 | 41,895 | + | asu-miR-57-5p/ | *Ascaris suum* |
|  |  |  |  | aae-miR-993/ | *Aedes aegypti* |
|  |  |  |  | cte-miR-10a | *Capitella teleta* |
|  | 41,922 | 41,944 | + | MIN0027 | *Meloidogyne incognita* (this study) |
| MiV1ctg151 | 81,937 | 81,960 | − | MIN0158 | *Meloidogyne incognita* (this study) |
|  | 83,472 | 83,493 | + | MIN0419 | *Meloidogyne incognita* (this study) |
| MiV1ctg153 | 26,406 | 26,426 | + | MIN0275 | *Meloidogyne incognita* (this study) |
|  | 28,259 | 28,279 | − | ptr-miR-6130 | *Pan troglodytes* |
| MiV1ctg155 | 69,676 | 69,699 | + | MIN0455 | *Meloidogyne incognita* (this study) |
|  | 69,826 | 69,849 | + | MIN0352 | *Meloidogyne incognita* (this study) |
| MiV1ctg159 | 88,684 | 88,707 | + | MIN0116 | *Meloidogyne incognita* (this study) |
|  | 89,384 | 89,401 | − | bta-miR-2284f | *Bos taurus* |
| MiV1ctg161 | 28,615 | 28,635 | − | ptr-miR-3132 | *Pan troglodytes* |
|  | 29,176 | 29,199 | + | MIN0303 | *Meloidogyne incognita* (this study) |
| MiV1ctg162 | 44,780 | 44,801 | − | MIN0719 | *Meloidogyne incognita* (this study) |
|  | 44,832 | 44,854 | + | MIN0199 | *Meloidogyne incognita* (this study) |
| MiV1ctg164 | 25,002 | 25,179 | + | mdo-miR-7398d-5p | *Monodelphis domestica* |
| MiV1ctg167 | 84,274 | 84,291 | − | mml-miR-181b-2-3p | *Macaca mulatta* |
|  | 84,856 | 84,878 | + | MIN0206 | *Meloidogyne incognita* (this study) |
|  | 85,109 | 85,134 | − | cqu-miR-988-5p | *Culex quinquefasciatus* |
|  | 86,313 | 86,337 | − | mmu-miR-483-5p | *Mus musculus* |
|  | 86,371 | 86,391 | − | tca-miR-2944c-5p | *Tribolium castaneum* |
| MiV1ctg1691 | 126 | 149 | + | crm-miR-239b | *Caenorhabditis remanei* |
|  | 178 | 199 | + | MIN0103 | *Meloidogyne incognita* (this study) |
| MiV1ctg17 | 167,183 | 167,209 | + | bma-miR-228 | *Brugia malayi* |
|  | 168,044 | 168,064 | + | MIN0404 | *Meloidogyne incognita* (this study) |
| MiV1ctg172 | 64,117 | 64,138 | − | MIN0030 | *Meloidogyne incognita* (this study) |
|  | 64,842 | 64,863 | + | MIN0040 | *Meloidogyne incognita* (this study) |
| MiV1ctg18 | 227,683 | 227,703 | − | ptr-miR-3132 | *Pan troglodytes* |
|  | 228,239 | 228,262 | + | MIN0303 | *Meloidogyne incognita* (this study) |
| MiV1ctg181 | 62,265 | 62,286 | − | MIN0552 | *Meloidogyne incognita* (this study) |
| MiV1ctg192 | 49,628 | 49,646 | + | mmu-miR-1224-5p | *Mus musculus* |
|  | 49,688 | 49,707 | + | mmu-miR-2861 | *Mus musculus* |
|  | 60,691 | 60,710 | + | hsa-miR-1268a | *Homo sapiens* |
| MiV1ctg1924 | 3645 | 3,673 | + | hsa-miR-100-5p | *Homo sapiens* |
|  | 4091 | 4,116 | + | hsa-let-7c-5p | *Homo sapiens* |
|  | 4138 | 4,160 | + | MIN0270 | *Meloidogyne incognita* (this study) |
|  | 5744 | 5,766 | + | MIN0603 | *Meloidogyne incognita* (this study) |
| MiV1ctg1938 | 2084 | 2,105 | + | MIN0762 | *Meloidogyne incognita* (this study) |
|  | 2732 | 2,754 | + | MIN0666 | *Meloidogyne incognita* (this study) |
|  | 3959 | 3,980 | − | MIN0252 | *Meloidogyne incognita* (this study) |
|  | 5066 | 5,087 | − | rno-miR-466b-1-3p | *Rattus norvegicus* |
| MiV1ctg2 | 111,341 | 111,363 | − | MIN0017 | *Meloidogyne incognita* (this study) |
|  | 111,800 | 111,822 | + | MIN0092 | *Meloidogyne incognita* (this study) |
| MiV1ctg20 | 42,383 | 42,407 | + | MIN0718/ MIN0702 | *Meloidogyne incognita* (this study) |
|  | 42,448 | 42,469 | − | MIN0586 | *Meloidogyne incognita* (this study) |
|  | 43,317 | 43,449 | + | cel-miR-71-5p | *Caenorhabditis elegans* |
|  | 43,596 | 43,620 | + | cel-miR-2-3p | *Caenorhabditis elegans* |
| MiV1ctg209 | 24,055 | 24,076 | + | MIN0277 | *Meloidogyne incognita* (this study) |
|  | 24,898 | 24,921 | − | MIN0517 | *Meloidogyne incognita* (this study) |
| MiV1ctg2099 | 2475 | 2,497 | − | MIN0510 | *Meloidogyne incognita* (this study) |
|  | 3234 | 3,255 | − | MIN0362 | *Meloidogyne incognita* (this study) |
| MiV1ctg219 | 85,234 | 85,255 | − | oan-miR-219-3p | *Ornithorhynchus anatinus* |
|  | 86,829 | 86,851 | − | MIN0551 | *Meloidogyne incognita* (this study) |
| MiV1ctg22 | 61,706 | 61,728 | − | MIN0627 | *Meloidogyne incognita* (this study) |
|  | 154,670 | 154,692 | + | MIN0043 | *Meloidogyne incognita* (this study) |
|  | 154,975 | 154,992 | − | mmu-miR-1188-5p | *Mus musculus* |
|  | 167,917 | 167,940 | − | MIN0192 | *Meloidogyne incognita* (this study) |
|  | 169,260 | 169,282 | + | ame-miR-92c | *Apis mellifera* |
|  | 175,457 | 175,475 | − | sha-miR-200b | *Sarcophilus harrisii* |
| MiV1ctg220 | 66,966 | 66,988 | + | MIN0591 | *Meloidogyne incognita* (this study) |
| MiV1ctg221 | 69,289 | 69,313 | − | cel-miR-2-3p | *Caenorhabditis elegans* |
|  | 69,458 | 69,590 | − | cel-miR-71-5p | *Caenorhabditis elegans* |
|  | 70,461 | 70,483 | − | MIN0718 | *Meloidogyne incognita* (this study) |
| MiV1ctg227 | 82,596 | 82,615 | − | tca-miR-3851f-5p | *Tribolium castaneum* |
|  | 84,392 | 84,415 | + | MIN0287 | *Meloidogyne incognita* (this study) |
| MiV1ctg230 | 12,476 | 12,502 | − | asu-miR-92-3p/ | *Ascaris suum* |
|  |  |  |  | prd-miR-235-3p/ | *Panagrellus redivivus* |
|  |  |  |  | bbe-miR-2059-5p/ | *Branchiostoma belcheri* |
|  |  |  |  | dvi-miR-310 | *Drosophila virilis* |
|  | 12,527 | 12,549 | − | MIN0133 | *Meloidogyne incognita* (this study) |
|  | 14,298 | 14,319 | − | MIN0668 | *Meloidogyne incognita* (this study) |
| MiV1ctg237 | 58,458 | 58,479 | + | mmu-miR-466f-3p | *Mus musculus* |
| MiV1ctg255 | 20,535 | 20,557 | − | MIN0343 | *Meloidogyne incognita* (this study) |
|  | 20,822 | 20,844 | + | hsa-miR-25-5p | *Homo sapiens* |
| MiV1ctg256 | 76,392 | 76,414 | + | MIN0206 | *Meloidogyne incognita* (this study) |
|  | 76,647 | 76,672 | − | cqu-miR-988-5p | *Culex quinquefasciatus* |
|  | 77,851 | 77,875 | − | mmu-miR-483-5p | *Mus musculus* |
|  | 77,909 | 77,929 | − | tca-miR-2944c-5p | *Tribolium castaneum* |
| MiV1ctg268 | 23,918 | 24,004 | + | asu-miR-234-5p | *Ascaris suum* |
| MiV1ctg2865 | 1936 | 1,958 | + | MIN0631 | *Meloidogyne incognita* (this study) |
|  | 1989 | 2,011 | − | MIN0630 | *Meloidogyne incognita* (this study) |
|  | 1991 | 2,013 | + | MIN0020 | *Meloidogyne incognita* (this study) |
| MiV1ctg289 | 65,394 | 65,415 | − | hsa-miR-4520a-3p | *Homo sapiens* |
|  | 63,407 | 63,428 | + | MIN0621 | *Meloidogyne incognita* (this study) |
|  | 48,000 | 48,019 | − | MIN0620 | *Meloidogyne incognita* (this study) |
|  | 59,526 | 59,546 | − | MIN0426 | *Meloidogyne incognita* (this study) |
|  | 49,928 | 49,949 | − | MIN0209 | *Meloidogyne incognita* (this study) |
|  | 67,101 | 67,122 | − | hsa-miR-219a-2-3p | *Homo sapiens* |
| MiV1ctg310 | 45,466 | 45,593 | − | MIN0751 | *Meloidogyne incognita* (this study) |
| MiV1ctg313 | 68,799 | 68,822 | + | MIN0486 | *Meloidogyne incognita* (this study) |
|  | 69,492 | 69,512 | + | tca-miR-3851f-5p | *Tribolium castaneum* |
| MiV1ctg33 | 185,527 | 185,551 | + | aca-miR-99b-5p/ | *Anolis carolinensis* |
|  |  |  |  | bfl-miR-100-5p/ | *Branchiostoma floridae* |
|  |  |  |  | dre-miR-10a-5p/ | *Danio rerio* |
|  |  |  |  | cel-miR-57-5p | *Caenorhabditis elegans* |
|  | 185,600 | 185,622 | + | MIN0078 | *Meloidogyne incognita* (this study) |
| MiV1ctg34 | 8433 | 8,456 | − | MIN0404 | *Meloidogyne incognita* (this study) |
|  | 9276 | 9,302 | − | bma-miR-228 | *Brugia malayi* |
| MiV1ctg342 | 11,488 | 11,574 | + | asu-miR-234-5p | Ascaris suum |
| MiV1ctg346 | 30,081 | 30,101 | + | MIN0007 | *Meloidogyne incognita* (this study) |
|  | 31,742 | 31,765 | + | MIN0472 | *Meloidogyne incognita* (this study) |
| MiV1ctg361 | 24,418 | 24,439 | + | rno-miR-466b-1-3p | *Rattus norvegicus* |
|  | 26,185 | 26,206 | − | MIN0333 | *Meloidogyne incognita* (this study) |
| MiV1ctg384 | 43,585 | 43,606 | − | MIN0040 | *Meloidogyne incognita* (this study) |
|  | 44,310 | 44,331 | + | MIN0030 | *Meloidogyne incognita* (this study) |
| MiV1ctg387 | 53,738 | 53,760 | + | MIN0017 | *Meloidogyne incognita* (this study) |
|  | 53,856 | 53,878 | − | MIN0092 | *Meloidogyne incognita* (this study) |
| MiV1ctg394 | 26,272 | 26,294 | + | MIN0636 | *Meloidogyne incognita* (this study) |
|  | 26,295 | 26,318 | − | MIN0481 | *Meloidogyne incognita* (this study) |
|  | 26,361 | 26,383 | − | MIN0319 | *Meloidogyne incognita* (this study) |
|  | 28,325 | 28,347 | − | MIN0665 | *Meloidogyne incognita* (this study) |
| MiV1ctg42 | 107,915 | 107,937 | − | MIN0188 | *Meloidogyne incognita* (this study) |
|  | 108,677 | 108,700 | − | MIN0198 | *Meloidogyne incognita* (this study) |
| MiV1ctg422 | 2729 | 2,752 | − | dre-miR-22b | *Danio rerio* |
|  | 2730 | 2,753 | − | MIN0217 | *Meloidogyne incognita* (this study) |
|  | 3419 | 3,440 | + | MIN0149 | *Meloidogyne incognita* (this study) |
| MiV1ctg430 | 47,614 | 47,634 | − | mmu-miR-1224-5p | *Mus musculus* |
|  | 47,721 | 47,739 | − | mmu-miR-2861 | *Mus musculus* |
| MiV1ctg451 | 19,822 | 19,842 | + | MIN0261 | *Meloidogyne incognita* (this study) |
|  | 21,224 | 21,246 | − | mml-miR-3145-3p | *Macaca mulatta* |
| MiV1ctg47 | 170,763 | 170,784 | − | MIN0682 | *Meloidogyne incognita* (this study) |
|  | 171,345 | 171,365 | − | MIN0358 | *Meloidogyne incognita* (this study) |
| MiV1ctg5 | 267,191 | 267,212 | + | cfa-miR-329a | *Canis familiaris* |
|  | 267,437 | 267,458 | + | MIN0177 | *Meloidogyne incognita* (this study) |
|  | 270,139 | 270,161 | + | pma-miR-190b-5p | *Petromyzon marinus* |
|  | 270,301 | 270,322 | + | MIN0656 | *Meloidogyne incognita* (this study) |
| MiV1ctg50 | 150,635 | 150,655 | + | ptr-miR-6130 | *Pan troglodytes* |
|  | 152,485 | 152,505 | − | MIN0275 | *Meloidogyne incognita* (this study) |
| MiV1ctg516 | 24,781 | 24,798 | + | hsa-miR-3622b-5p | *Homo sapiens* |
|  | 25,230 | 25,251 | + | hsa-miR-515-3p | *Homo sapiens* |
| MiV1ctg53 | 74,302 | 74,324 | + | MIN0350 | *Meloidogyne incognita* (this study) |
|  | 74,732 | 74,751 | + | hsa-miR-2392 | *Homo sapiens* |
| MiV1ctg547 | 11,747 | 11,769 | − | MIN0398 | *Meloidogyne incognita* (this study) |
|  | 12,367 | 12,389 | + | MIN0114 | *Meloidogyne incognita* (this study) |
| MiV1ctg552 | 4644 | 4,666 | + | hsa-miR-548m | *Homo sapiens* |
|  | 5061 | 5,083 | − | hsa-miR-553 | *Homo sapiens* |
|  | 5642 | 5,663 | − | MIN0337 | *Meloidogyne incognita* (this study) |
| MiV1ctg554 | 34,722 | 34,743 | + | MIN0058 | *Meloidogyne incognita* (this study) |
|  | 35,933 | 35,954 | − | MIN0645 | *Meloidogyne incognita* (this study) |
| MiV1ctg614 | 19,086 | 19,108 | + | MIN0770 | *Meloidogyne incognita* (this study) |
|  | 19,138 | 19,159 | − | MIN0237 | *Meloidogyne incognita* (this study) |
| MiV1ctg638 | 37,469 | 37,618 | + | MIN0720 | *Meloidogyne incognita* (this study) |
| MiV1ctg644 | 18,859 | 18,880 | − | MIN0112 | *Meloidogyne incognita* (this study) |
|  | 18,954 | 18,979 | − | dme-miR-279-3p | *Drosophila melanogaster* |
|  | 19,008 | 19,030 | − | MIN0187 | *Meloidogyne incognita* (this study) |
| MiV1ctg655 | 32,120 | 32,142 | + | MIN0570 | *Meloidogyne incognita* (this study) |
|  | 32,904 | 32,924 | + | MIN0155 | *Meloidogyne incognita* (this study) |
| MiV1ctg677 | 31,553 | 31,575 | − | MIN0743 | *Meloidogyne incognita* (this study) |
|  | 33,165 | 33,187 | + | MIN0723 | *Meloidogyne incognita* (this study) |
|  | 33,234 | 33,252 | + | sme-miR-124e-5p | *Schmidtea mediterranea* |
| MiV1ctg687 | 33,204 | 33,226 | − | MIN0638 | *Meloidogyne incognita* (this study) |
|  | 33,418 | 33,440 | − | MIN0086 | *Meloidogyne incognita* (this study) |
| MiV1ctg7 | 176,234 | 176,254 | + | MIN0227 | *Meloidogyne incognita* (this study) |
|  | 177,333 | 177,354 | + | MIN0567 | *Meloidogyne incognita* (this study) |
| MiV1ctg726 | 22,611 | 22,634 | − | MIN0351 | *Meloidogyne incognita* (this study) |
|  | 24,144 | 24,166 | − | MIN0180 | *Meloidogyne incognita* (this study) |
| MiV1ctg739 | 28,407 | 28,430 | + | hsa-miR-1294 | *Homo sapiens* |
|  | 29,533 | 29,556 | − | MIN0375 | *Meloidogyne incognita* (this study) |
| MiV1ctg75 | 109,253 | 109,276 | + | MIN0455 | *Meloidogyne incognita* (this study) |
|  | 109,403 | 109,426 | + | MIN0352 | *Meloidogyne incognita* (this study) |
| MiV1ctg78 | 50,139 | 68,445 | − | hsa-miR-3622b-5p | *Homo sapiens* |
|  | 51,709 | 70,031 | + | hsa-miR-676-5p | *Homo sapiens* |
| MiV1ctg781 | 16,033 | 16,052 | + | MIN0173 | *Meloidogyne incognita* (this study) |
| MiV1ctg809 | 3676 | 3,699 | − | mmu-miR-466i-5p | *Mus musculus* |
|  |  |  | + | rno-miR-466b-1-3p | *Rattus norvegicus* |
|  | 3985 | 4,006 | + | MIN0413 | *Meloidogyne incognita* (this study) |
|  | 4029 | 4,049 | + | MIN0629 | *Meloidogyne incognita* (this study) |
| MiV1ctg86 | 15,185 | 15,207 | + | dre-miR-22b | *Danio rerio* |
|  | 14,454 | 14,477 | − | MIN0217 | *Meloidogyne incognita* (this study) |
|  | 15,138 | 15,159 | + | MIN0150 | *Meloidogyne incognita* (this study) |
| MiV1ctg876 | 10,111 | 10,133 | + | MIN0501 | *Meloidogyne incognita* (this study) |
|  | 11,791 | 11,814 | + | MIN0634 | *Meloidogyne incognita* (this study) |
| MiV1ctg88 | 121,301 | 121,325 | + | aca-miR-99b-5p | *Anolis carolinensis* |
|  |  |  |  | bfl-miR-100-5p | *Branchiostoma floridae* |
|  |  |  |  | dre-miR-10a-5p | *Danio rerio* |
|  |  |  |  | cel-miR-57-5p | *Caenorhabditis elegans* |
|  | 121,374 | 121,396 | + | MIN0078 | *Meloidogyne incognita* (this study) |
| MiV1ctg886 | 9309 | 9,331 | + | hsa-miR-548m | *Homo sapiens* |
|  | 9726 | 9,748 | − | hsa-miR-553 | *Homo sapiens* |
|  | 10,307 | 10,328 | − | MIN0337 | *Meloidogyne incognita* (this study) |
| MiV1ctg889 | 12,209 | 12,232 | − | MIN0116 | *Meloidogyne incognita* (this study) |
| MiV1ctg9 | 133,529 | 133,550 | + | MIN0594 | *Meloidogyne incognita* (this study) |
|  | 133,572 | 133,593 | − | MIN0672 | *Meloidogyne incognita* (this study) |
|  | 134,249 | 134,271 | − | MIN0371 | *Meloidogyne incognita* (this study) |
| MiV1ctg90 | 42,210 | 42,232 | − | MIN0027 | *Meloidogyne incognita* (this study) |
|  | 42,266 | 42,290 | − | dme-miR-993-5p | *Drosophila melanogaster* |
|  |  |  |  | cte-miR-10a | *Capitella teleta* |
|  |  |  |  | asu-miR-57-5p | *Ascaris suum* |
| MiV1ctg902 | 11,809 | 11,830 | + | MIN0209 | *Meloidogyne incognita* (this study) |
|  | 13,739 | 13,761 | + | MIN0620 | *Meloidogyne incognita* (this study) |
|  | 15,726 | 15,747 | − | hsa-miR-4520a-3p | *Homo sapiens* |
|  | 16,520 | 16,541 | − | MIN0577 | *Meloidogyne incognita* (this study) |
|  | 17,433 | 17,454 | − | hsa-miR-219a-2-3p | *Homo sapiens* |
| MiV1ctg97 | 43,856 | 43,877 | + | hsa-miR-219a-2-3p | *Homo sapiens* |
|  | 45,563 | 45,584 | + | hsa-miR-4520a-3p | *Homo sapiens* |
|  | 47,549 | 47,571 | − | MIN0621 | *Meloidogyne incognita* (this study) |
| MiV1ctg98 | 7391 | 7,414 | + | MIN0356 | *Meloidogyne incognita* (this study) |
|  | 7774 | 7,797 | + | dps-miR-iab-8-3p | *Drosophila pseudoobscura* |
|  | 26,136 | 26,158 | − | MIN0214 | *Meloidogyne incognita* (this study) |
|  | 26,138 | 26,160 | − | MIN0360 | *Meloidogyne incognita* (this study) |
|  | 26,196 | 26,218 | + | MIN0104 | *Meloidogyne incognita* (this study) |

**Table S8.** Mapping statistics of known and novel miRNA to the reference genome at Wormbase (ASM18041v1a).

| **Region** | **Known miRNA** | **Novel miRNA** |
| --- | --- | --- |
| Intergenic | 716 | 247 |
| Exon | 1 | 0 |
| Intron | 1430 | 217 |
| 3′ UTR | 0 | 0 |
| 5′ UTR | 0 | 0 |

**Table S9.** Known and novel miRNA of *M. incognita* common expressed in all stages of the nematode.

**Table S10.** Stage-specific Known and novel miRNA expressed in nematode *M. incognita.*

**Table S11.** Deseq2 normalization of known miRNA (*R* > 10).

**Table S12.** Deseq2 normalization of novel miRNA (*R* > 10).

**Table S13.** List of primers used for experimental validation of miRNA expression.

| **Stage** | **miRNA** | **miRBase Match** | **Sequence (5′ to 3′)** |
| --- | --- | --- | --- |
| Egg | MI00171 | miR-6053 | AACGAAGACCGCGGCGGAGCTGT |
|  | MI01750 | miR-2198 | GAGCTCGGTCGAAGGCGCCT |
|  | MI01855 | miR-5908-5p | GATGAGTTGTTGACGGTGAGG |
|  | MI01895 | miR-211-3p | GCAAGGACAGCAACATGGAGG |
|  | MI03228 | miR-7289-5p | TGGAGCAGTTGGGAGCTATGAG |
|  | MIN00267 | – | TAATTTTTGCCTGTAGCGGCTC |
|  | MIN00067 | – | AGCCCAATGTACCGTCGAGCTAT |
|  | MIN00192 | – | GGACCAGTTCCGATTTTCGGCT |
|  | MIN00038 | – | AATCTTACAACATTCCAGCATTC |
|  | MIN00371 | – | TTTGAAACTGAAGCAAATTTGC |
| J2 | MI01348 | miR-8089 | CATGGGGAATGGGATGGGGCAG |
|  | MI02028 | miR-2486-5p | GGAGAAGACGGGGGTGGTGGTGGG |
|  | MI02073 | miR-6627-3p | GGATGAAGACGATGATGCTGAGGA |
|  | MI03049 | miR-6922-5p | TGAGGGAGGGCATGTAGAGACGG |
|  | MI03464 | miR-46-3p | TGTCCTTTGGAGGTCGCTCTTTCA |
|  | MIN00235 | – | GTTCTTGATCGTTGACGCAACCAC |
|  | MIN00190 | – | GCTCTCGGCGCTGTGGGAGGCC |
|  | MIN00310 | – | TGAGCACTGTGTCCGAAGTTCCT |
|  | MIN00301 | – | TCTGGTTTGTAGTTAGAATGTG |
| J3 | MI00131 | miR-6949-5p | AAATGGGTGTGGAAAGAGGTCGAA |
|  | MI00880 | miR-6883-5p | AGGGGGAGGTGGTAGTGGATGT |
|  | MI02049 | miR-6923-5p | GGAGGAGGAGGAGGATTGGGTGG |
|  | MI02411 | miR-6607-5p | GTTGGTGGAGGAAGTGGAGGTGG |
|  | MIN00039 | – | AATGAAATTTTTGGAACTGGGGGT |
| J4 | MI01617 | miR-4830b-5p | CTTTGAGTCGATTGGAGGA |
|  | MI01808 | miR-7195-5p | GAGGGAGAAGGGGATGTTGAC |
|  | MI02241 | miR-5859 | GTAGAATTGTTGGTGGATGGCTT |
|  | MI03255 | miR-504-3p | TGGAGGTGCAGGGCGAGGTTTTC |
|  | MI03615 | miR-1497f | TTGAAGAGGATTGAAGGGGAGGG |
|  | MIN00172 | – | GATAGATAGTTGGCTGTTTCGGTT |
|  | MIN00022 | – | AAGGAAATTGGATGCCGGCATT |
| Female | MI00284 | miR-1547-3p | AAGGATCGCCTGATTTGGTCAT |
|  | MI01064 | miR-2024f-5p | ATGAAGAATGGTATGGGCAAAA |
|  | MI01792 | miR-5364 | GAGGATTGTTATTGGGCTGA |
|  | MI02695 | miR-274-5p | TATCGTGACGGACATAACGGGATA |
|  | MI03768 | miR-7336-5p | TTTGGATGAAAGTGTGGATG |
|  | MIN00184 | – | GCACTTCTCGGTCGTTGGCGAT |
|  | MIN00365 | – | TTGTATCGTCATAAGCATTTCGCA |
|  | MIN00102 | – | ATGCACATGGATACGGATTTTGC |
|  | MIN00193 | – | GGAGATCCTGGCTGTCCGACATG |

**Table S14.** List of commonly expressed candidate miRNAs chosen to be tested as internal standards for quantitative PCR (qPCR).

| **Given Name** | **miRNA** | **miRBASE Reference** | **Sequence** |
| --- | --- | --- | --- |
| MI03018 | miR-58-3p | prd-miR-58-3p | TGAGATCAGTCCAGATTCGT |
| MI02743 | miR-7904-3p | prd-miR-7904-3p | TCAAAAATTCCGTTGCGTCGCA |
| MI02950 | miR-71-5p | cel-miR-71-5p | TGAAAGACATGGGTAGTTGAGACG |

| **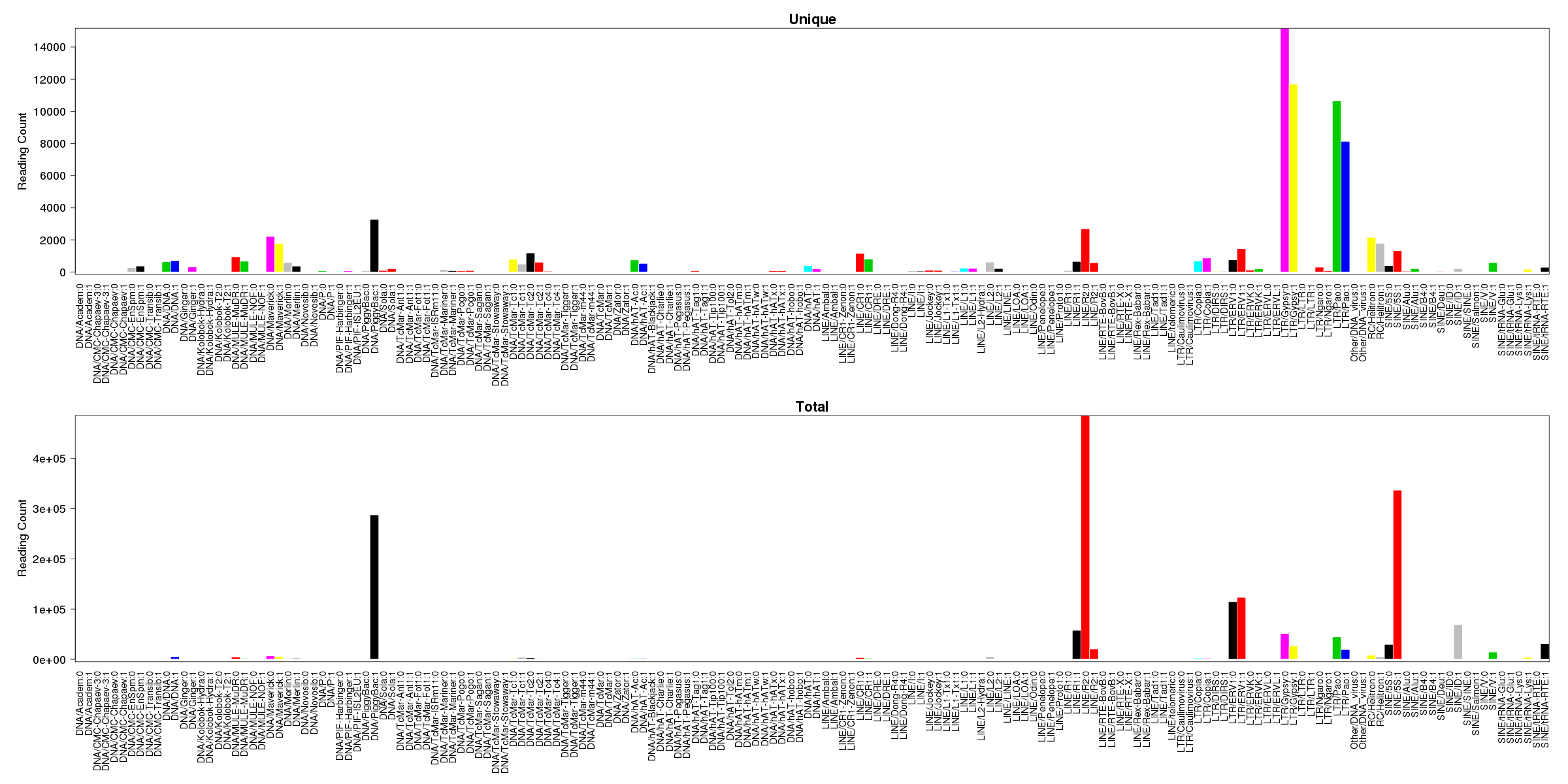** |
| --- |
| (**A**) |
| 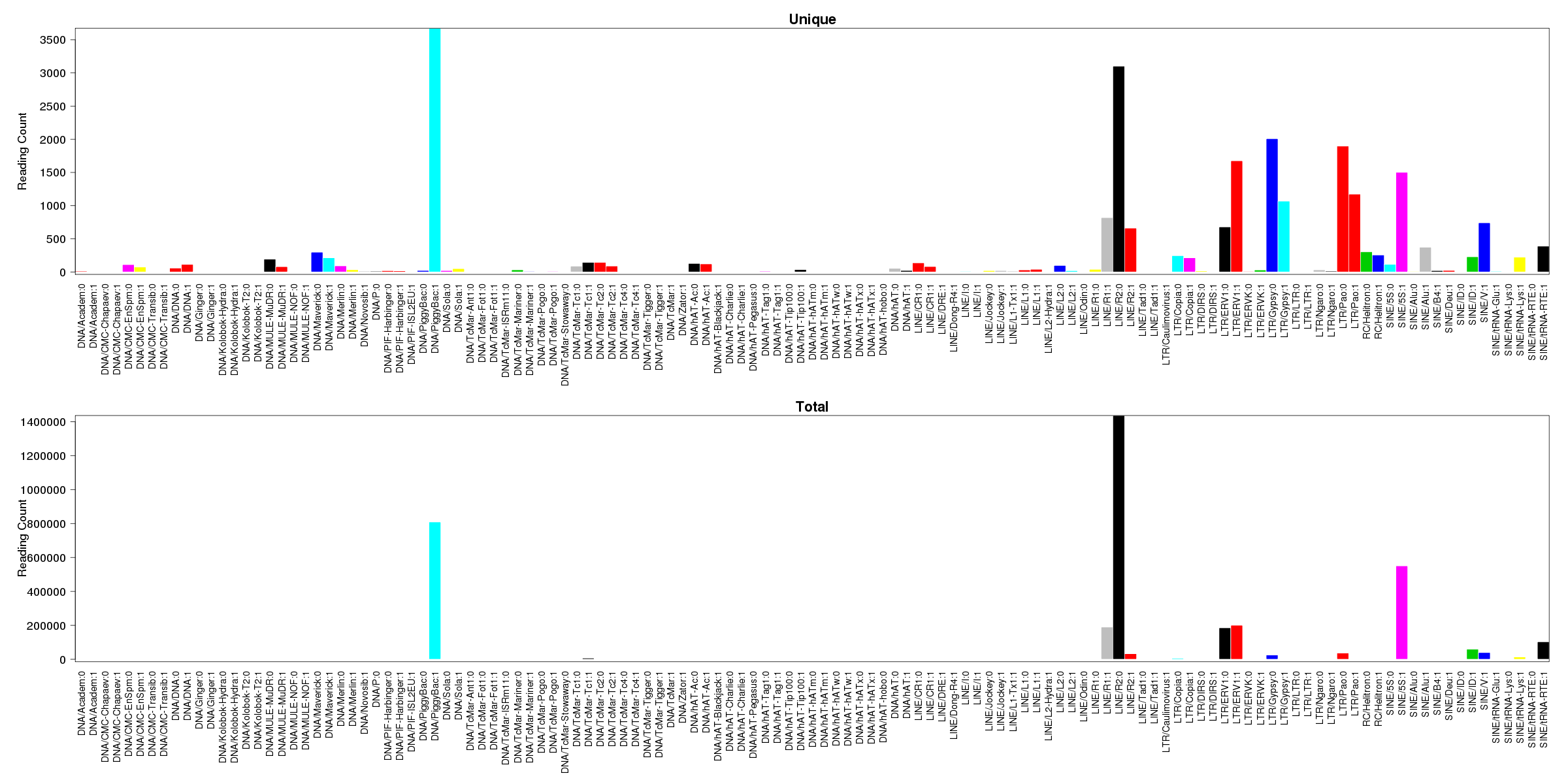 |
| (**B**) |
| 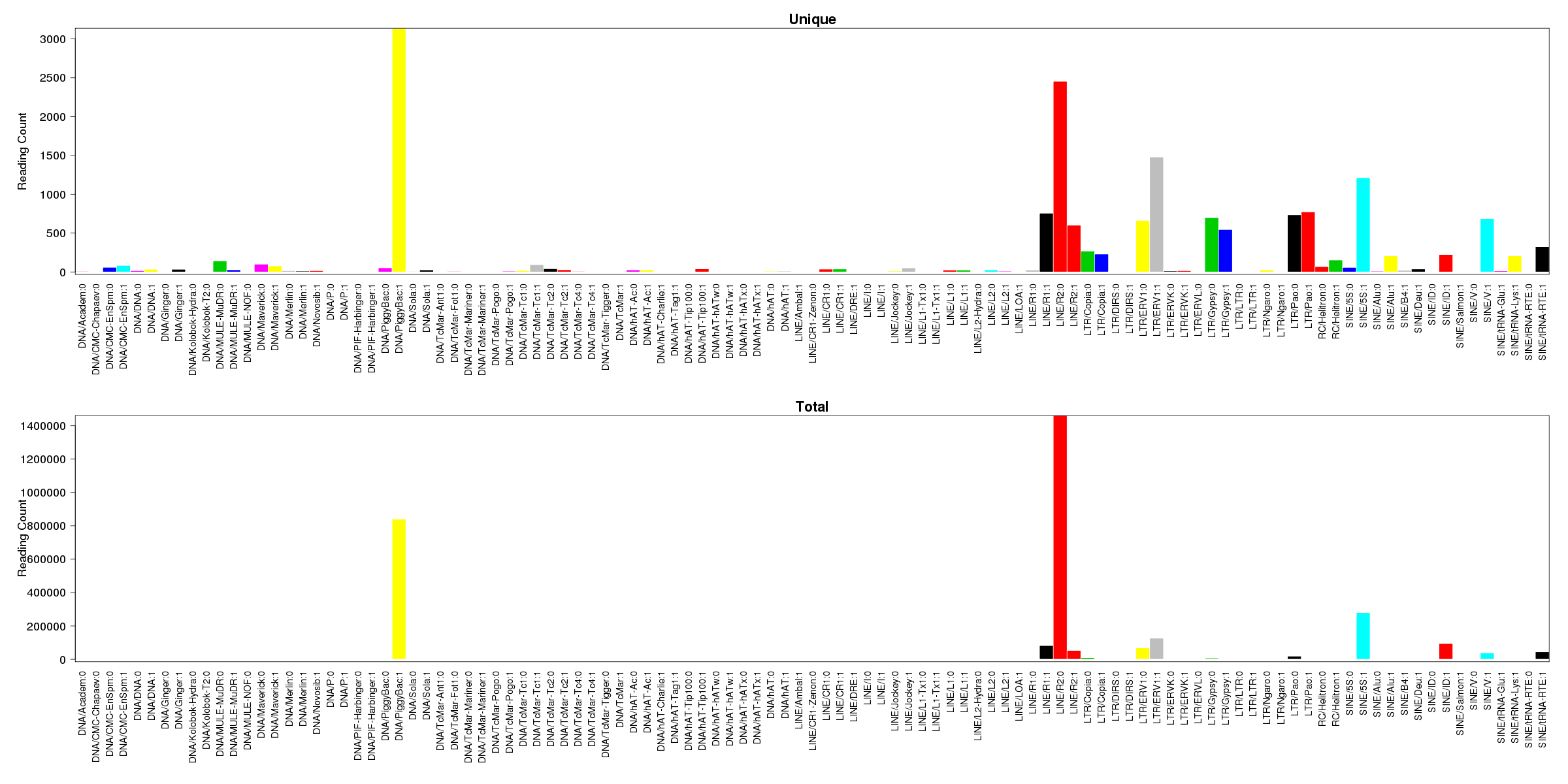 |
| (**C**) |
| 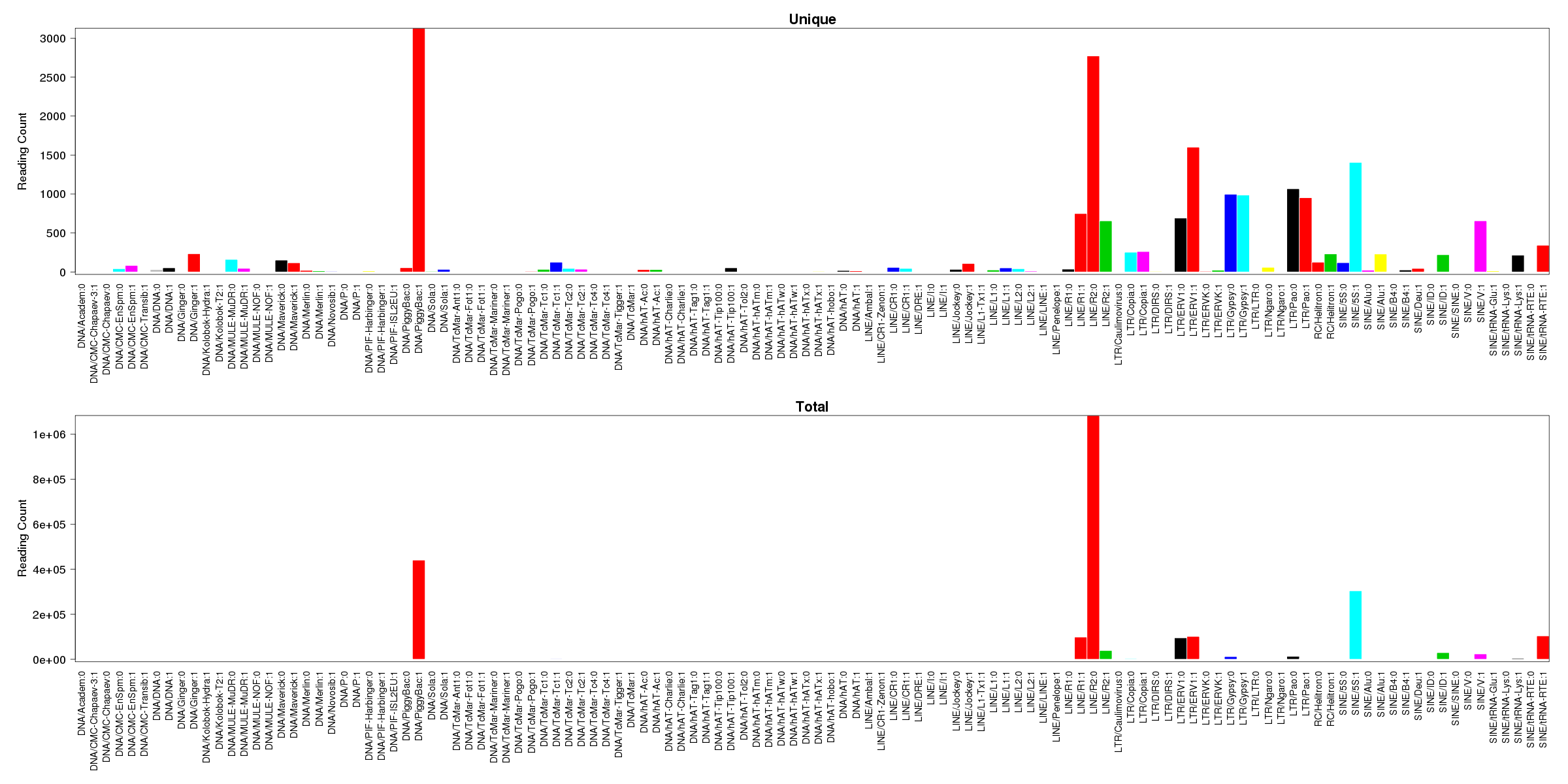 |
| (**D**) |
| 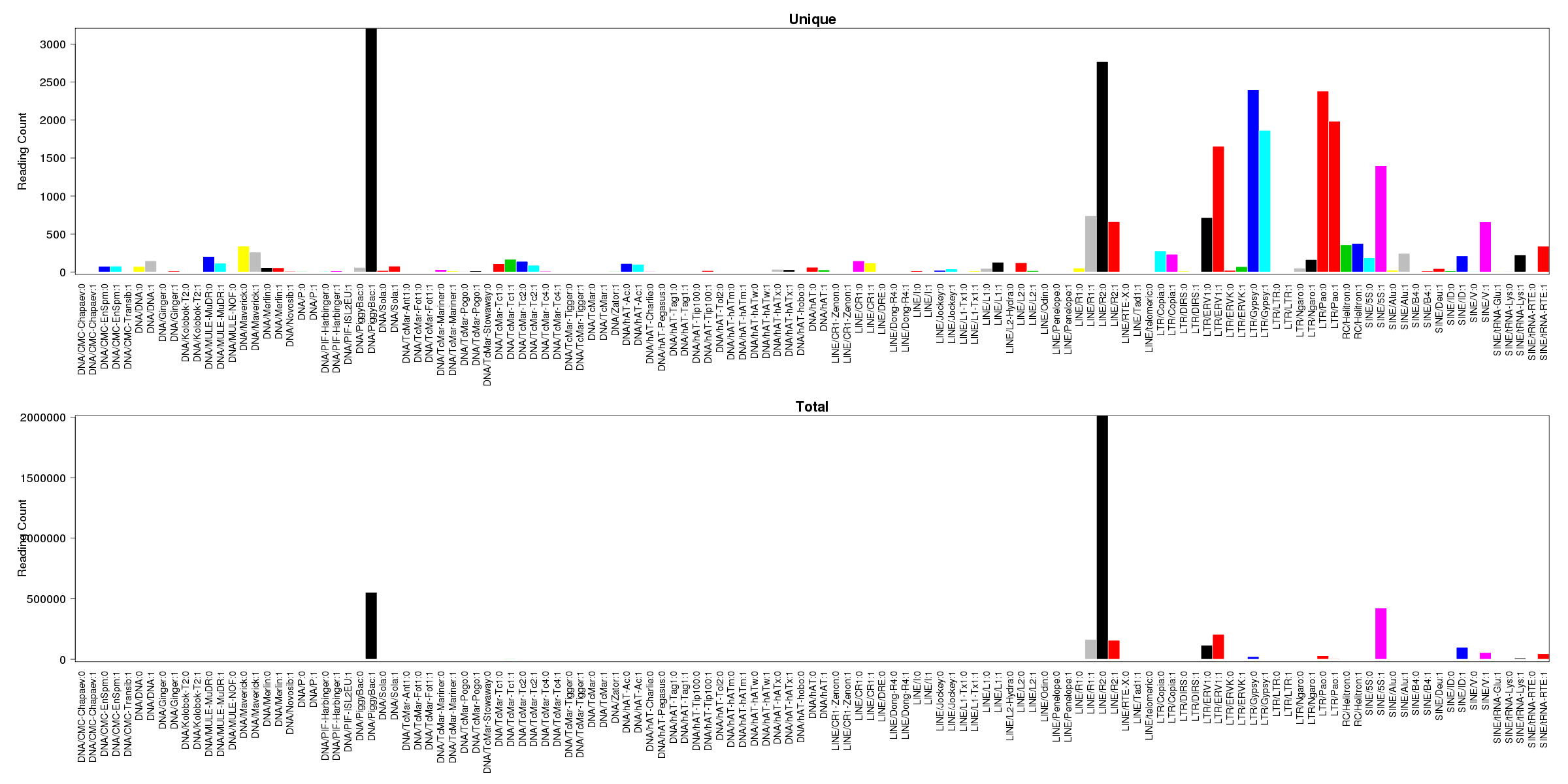 |
| (**E**) |

**Figure S1.** Distribution of unique and total repeat associated RNA. *x* axis is type of repeat associated sequence and *y* axis is number of a certain type of reads. (**A**) Egg; (**B**) J2; (**C**) J3; (**D**) J4; and (**E**) female stages.


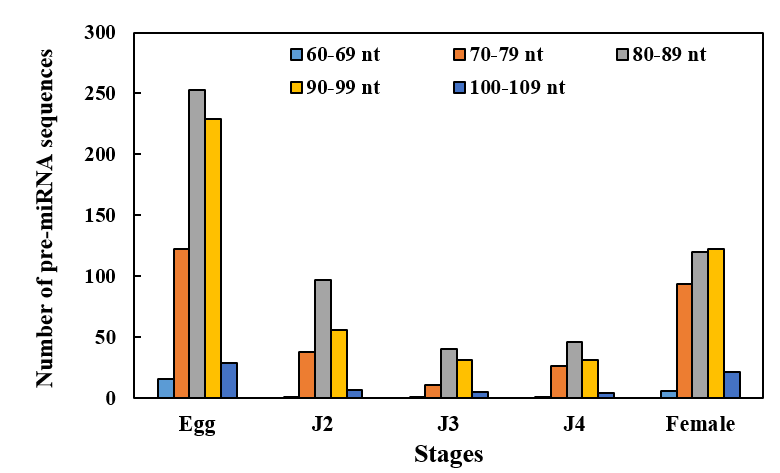


**Figure S2.** Size distribution of pre-miRNAs among different stages of *M. incognita.* Pre-miRNAs of varying length were obtained at stage of the nematode life cycle.


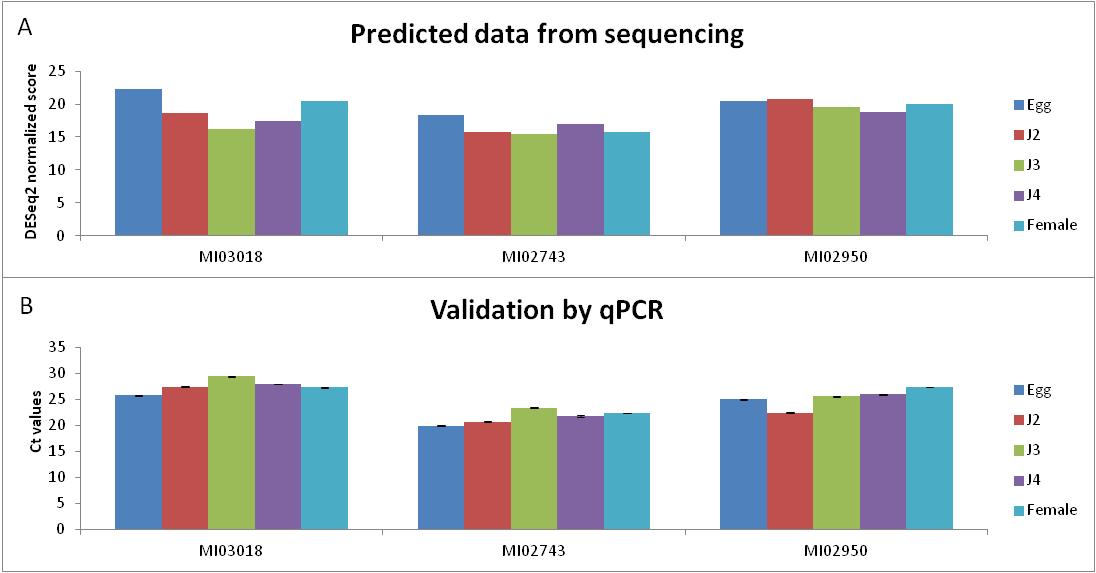


**Figure S3.** Validation of internal standards. A. DESeq2 normalized scores obtained for the candidate miRNAs using deep sequencing. B. Real time expression of candidate miRNA internal standards at various stages of *M. incognita* life cycle.


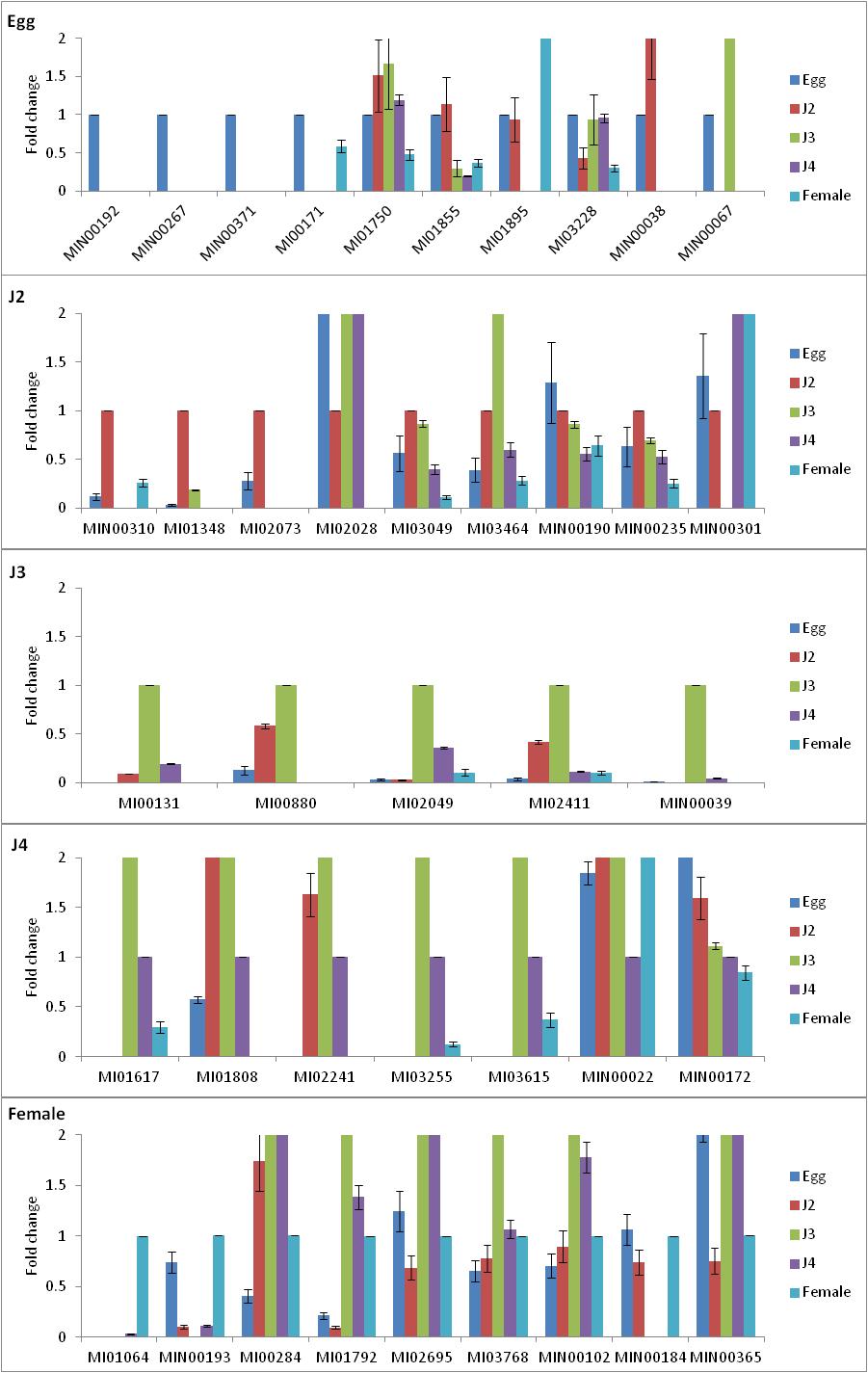


**Figure S4.** Real time expression of 40 miRNA across various stages of *M. incognita* life cycle studies using qPCR. Common miRNA MI03018 was used as an internal standard to compare expression.
